# Fto-mediated m^6^A modification is essential for cerebellar development through regulating epigenetic reprogramming

**DOI:** 10.1186/s12929-025-01176-0

**Published:** 2025-08-29

**Authors:** Jing Jiang, Ming Zhang, Wenjuan Xia, Chenyue Ding, Jincheng Li, Xiujuan Hu, Jiafeng Lu, Hong Li, Qingxia Meng, Hoi-Hung Cheung, Boxian Huang

**Affiliations:** 1 State Key Laboratory of Reproductive Medicine and Offspring Health (Suzhou Centre), Suzhou Municipal Hospital, Gusu School, Suzhou Affiliated Hospital of Nanjing Medical University, Nanjing Medical University, Suzhou, 215002 China; 2 School of Biomedical Sciences, Faculty of Medicine, The Chinese University of Hong Kong, Hong Kong, 999077 China

**Keywords:** Keywords, Cerebellar development, *Fto*, m^6^A modification, H4K16ac, *Kat8*

## Abstract

**Background:**

Growing evidence highlights the importance of epitranscriptomic regulation in cerebellar development and function, especially through m^6^A methylation. Nevertheless, the precise function of the RNA demethylase Fto in the cerebellum is still uncertain.

**Methods:**

An *Fto* knockout (*Fto*^*KO*^) mouse model was generated to investigate the role of *Fto* in cerebellar development. Cerebellar function was assessed using the behavioral tests and Nissl staining. Immunofluorescence was performed to detect molecular expression levels and subcellular localization. Dot blot, m^6^A-RIP-seq, ATAC-seq and CUT&Tag-seq were used to confirm m^6^A levels and chromatin accessibility. Co-IP was employed to test molecular interactions.

**Results:**

*Fto*^*KO*^ mice exhibited cerebellar ataxia, including tremors and abnormal gait patterns. Reduced FTO expression at embryonic day 13.5 (E13.5) and postnatal day 3 (P3) stages resulted in increased TUJ1 expression, as well as reductions in neuronal functional genes (*Map2*) and self-renewal genes (*Sox2*, *Sox9*, *Nestin* and *Pax6*). Mechanistically, *Kat8* upregulation was linked to the high m^6^A levels regulated by *Fto* loss. Furthermore, IGF2BP3 specifically recruited acetyltransferase KAT8 to control gene transcription during early cerebellar development by regulating H4K16ac modification, which alters chromatin accessibility in neural developmental pathways.

**Conclusions:**

In summary, *Fto*^*KO*^-induced *Kat8* upregulation in an m^6^A-dependent manner resulted in enhanced KAT8 recruitment by IGF2BP3, which improved chromatin accessibility and H4K16ac modification, thereby promoting cerebellar developmental dysfunction.

**Supplementary Information:**

The online version contains supplementary material available at 10.1186/s12929-025-01176-0.

## Background

The cerebellum is traditionally recognized for its role in motor control, but it is also known to contribute significantly to various cognitive functions through its connections with the cerebral cortex [1]. Despite constituting just 10% of the brain's mass, the cerebellum harbors a diverse array of neuron types that represent over 80% of the total neuronal population in the human brain [2]. The cerebellar cortex's architecture has remained remarkably consistent throughout evolution, with almost all mammals possessing three distinct layers: the molecular layer (ML), the Purkinje cell layer (PCL), and the granule cell layer (GCL) [3]. Interneurons interwoven within these layers facilitate cerebellar signal transduction [4]. This conserved structure renders the cerebellum a valuable model for studying neuronal cell differentiation, migration, and proliferation [5]. Pathological conditions affecting the cerebellum are frequently linked to disorders such as spinocerebellar ataxia [6], intellectual disabilities [7] and autism spectrum symptom [8]. Significant advancements have been achieved in elucidating the epigenetic mechanisms underlying cerebellar development, including DNA 5-hydroxymethylation [9], chromatin accessibility [10], and *N*6-methyladenosine (m^6^A) modifications [11]. Despite these findings, the precise role of RNA methylation in the mouse cerebellum during prenatal cerebellar development has yet to be completely understood.

FTO is identified at the molecular level as an RNA demethylase whose expression is inversely linked with the levels of m^6^A and 6 mA [12, 13]. Additionally, FTO is also thought to be associated with DNA 6 mA modifications [12, 13]. FTO is localized in both the nucleus and cytoplasm [12], with a mobile fraction that shuttles between these cellular compartments, potentially facilitated by its interaction with gene regulation [14]. Traditionally, FTO is thought to be essential for energy homeostasis and adipogenesis [15]. Recent findings indicate that FTO protein is highly abundant in the human brain and is associated with reduced brain volume in both adolescents and older adults [16]. Several neurological functions, including adult neurogenesis in the hippocampus and memory generation, have been shown to depend on FTO [17].

In addition to FTO, IGF2BP3, as a key m^6^A reader, plays a fundamental role in neural development and organogenesis [18]. Recent reports have highlighted an interaction between m^6^A modification and histones within the nervous system. For example, METTL14 knockout in neural stem cells (NSCs) causes raised general histone modifications. This is a result of m^6^A changes influencing the stability of transcripts encoding histone-modifying enzymes, which in turn leads to alterations in gene expression and cellular phenotypes [19]. These results notwithstanding, research on the relationships between m^6^A, particularly its demethylase FTO, and DNA epigenetics, specifically histone acetylation, are still scarce, especially in cerebellar development.

H4K16 acetylation is mediated by the histone acetyltransferase KAT8, also referred to as MOF or MYST1 [20]. KAT8 serves as a catalytic subunit in two distinct protein complexes: MSL (male-specific lethal) and NSL (non-specific lethal) [21]. Within the MSL complex, KAT8 is responsible for the majority of H4K16 acetylation [22]. This modification is widely applied to the male X chromosome by MSL, resulting in increased transcriptional elongation [23]. Furthermore, H4K16 acetylation is depleted at gene promoters while being enriched at L1 and LTR elements, thereby activating transcription within the L1 and LTR regions of transposable elements (TEs) [24]. Studies have shown that mutations in the human *KAT8* gene impair H4K16 acetylation, leading to developmental delays in fine motor skills, intellectual disabilities, and abnormal brain development [25]. Mice with brain-specific knockout of the *Kat8* gene exhibit inhibition in neuronal differentiation, reduced neural progenitor cells, and abnormal neuronal migration, all of which are associated with lower levels of H4K16 acetylation [25]. However, the interaction between m^6^A modifications on KAT8 and histone acetylation, notably H4K16 acetylation in cerebellar development remains an intriguing and unresolved scientific question.

This paper aims to clarify the primary regulatory functions of FTO in the development of the prenatal mouse cerebellum, specifically examining its interactions with histone acetylation. We demonstrate that a reduction in the m^6^A eraser FTO, in conjunction with recognition effect by the m^6^A reader IGF2BP3, enhances the m^6^A-mRNA modification levels of *Kat8*. This augmentation results in elevated levels of H4K16ac, improved chromatin accessibility, and transcriptional activation.

## Methods

### Generation of *Fto* knockout mice

Mouse strains used in this study were C57BL/6. C57BL/6 mice were maintained in the Animal Research Center of Nanjing Medical University. *Fto*^*KO*^ mice were generated by intercrossing *Fto* heterozygous mice. All mouse pups were genotyped to identify the *Fto* deletion using tail DNA extract. All sequences of primers for genotyping are listed in Table S1.

### Tail suspension test

The tail-suspension test (TST) involves suspending mice by their tails above the ground. Mice at 8 weeks old were hung by the tail. Stick the tape firmly on the mouse tail and suspension rod. The test typically lasts 6 min. Remove the tape after the experiment is over. During this test, the postures and movements of the mice were observed. When the mice were suspended by their tails, their hind limbs remained in a crossed position indicating the presence of cerebellar ataxia.

### Footprint test

The hind paws and forepaws of mice were painted with nontoxic blue watercolor respectively. Mice walked through a tunnel (60 cm long, 10 cm wide) with white paper at the bottom. Each mouse was trained for three consecutive days with three trials per day. Footprint patterns were analyzed for length of stride, stance, sway and distance between the front and hind footprints on each side. If the mouse stopped in the middle of the tunnel, the trial was repeated.

### Rotarod test

The rotarod test is a behavioral test of motor coordination in mice by measuring the time spent staying and running on an accelerated rotarod. After the habituation to rotarod, mice (2 month) were tested twice a day. The velocity of rotation increased at a constant acceleration of 40 rpm/min starting from 4 rpm and the spent time on the rotarod was measured. The recovery period between trials was 15 min.

### Mouse cerebellar granule neuron precursors isolation and culturing

Cerebellar granule neuron precursors (GNPs) suspensions were prepared from 7-day-old wild-type mouse pups. Briefly, cerebellum from postnatal day 7 (P7) mice were dissected and washed by PBS (Gibco, C10010500CP) and incubated in 0.25% trypsin–EDTA (Gibco, 25200072) for 15 min at 37 °C. Trypsin digestion was terminated using complete media, and the cell suspensions were mechanically dissociated and filtered using a 100 μm cell strainer. Cells were centrifuged at 1000 rpm for 5 min. GNPs were plated on poly-L-lysine (Sangon Biotech, E607015) coated plates and cultured in mouse cerebellar granule cell complete culture medium (ZQXZBIO, PCM-M-83) in a 5% CO_2_, 37 °C incubator.

### Plasmids preparation and transfection

Plasmids containing overexpression *Fto* (wild-type) were constructed by cloning cDNA into the pcDNA3.1 expression vector (YouBio, VT8001). *Fto* (mutant; H231A and D233A) expression plasmid generated by site-directed mutagenesis (Vazyme, C215-01). Primer sequences are provided in Table S2. Cells were transfected using Lipofectamine 2000 reagent (Invitrogen, 11668019) according to the manufacturer’s instructions.

### Immunofluorescence analysis

Sagittal paraffin Sects. (5 μm) were cut and dewaxed, then blocked in 10% goat serum at room temperature (RT) for 1 h. After washing with PBS, sections were incubated with primary antibodies overnight at 4 °C and with secondary antibodies for 1 h at RT. Primary antibodies used in this study are provided in Table S3. Secondary antibodies, Alexa Fluor 488-conjugated goat anti-mouse IgG and Alexa Fluor 555-conjugated goat anti-rabbit IgG, were diluted at 1:500 in PBS. Cell nuclei were counterstained with 4,6-diamidino-2-phenylindole dihydrochloride (DAPI). Three pairs of wild type and *Fto* knockout mice were used for quantification. Imaging was taken with an Olympus BX53 fluorescence microscope and analyzed using Image J software.

### Nissl staining

Nissl staining was performed using Nissl staining solution (Servicebio, G1036). Sagittal cerebellar Sects. (5 μm) were dewaxed, immersed in Nissl staining solution for 4 min, and rinsed with PBS. The slides are sealed with neutral balsam mounting medium. Images of the cerebellum were captured using an Olympus microscope and analyzed by Image J software.

### TUNEL staining

Terminal uridine deoxynucleotidyl transferase (dUTP) nick end labeling (TUNEL) staining was performed using a commercial kit (In Situ Cell Death Detection Kit, TMR red; Roche, 12156792910) following the manufacturer’s instructions to detect apoptotic neurons in the cerebellar region. The cerebellar sections were rinsed three times in PBS and covered with 20 μg/ml proteinase K at 37 °C for 30 min, then washed with PBS to terminate enzymatic activity. They were then treated with a TUNEL reaction mixture containing 90% terminal deoxynucleotidyl transferase (TdT) buffer, 5% dUTP-biotin and 5% TdT in a humid dark box at 37 °C for 1 h. Sections were counterstained with DAPI. Tunel-positive cells in the cerebellar region were photographed with an Olympus BX53 fluorescence microscope and counted with Image J software.

### Western blot

The cerebellum tissues of E13.5 fetal mice was isolated under a stereomicroscope and homogenized using a freeze grinder. The pulverized tissue samples were lysed in RIPA buffer (Beyotime, P0013B) supplemented with phosphatase and protease inhibitor cocktails (Roche, 11873580001). The lysate was centrifuged at 12,000 rpm at 4 °C for 20 min to remove cell debris. The BCA Protein Assay Kit (Beyotime, P0012) was used to determine the protein concentration. Then, it was mixed with SDS-PAGE sample buffer before separation on a 12% SDS-PAGE gel. The sample protein was transferred to a PVDF membrane, blocked with TBST containing 5% skimmed milk, and detected with primary and secondary antibodies (see Table S3 for details). Protein signals were detected using an enhanced chemiluminescence kit and scanned.

### Co-immunoprecipitation and immunoblot assay

Cerebellar tissues (E13.5, P3 and 16w) were used for co-immunoprecipitation. Immunoprecipitation was performed according to a protocol from Roche Diagnostics. Protein A/G magnetic beads (MCE, HY-K0202) were washed three times with lysis buffer and incubated with 400 μg of tissue lysate together with 4 μg of rabbit anti-FTO, rabbit anti-KAT8, rabbit anti-IGF2BP3 or IgG overnight at 4 °C. Beads were washed six times with cold wash buffer (PBS containing 0.5% Tween-20). The eluate was neutralized with 5 × SDS sample buffer and briefly heated at 100 °C before being used for western blot analysis.

### RNA isolation

The pregnant mice were sacrificed by cervical dislocation, and the fetal cerebellum was immediately removed and flash frozen in liquid nitrogen. Total RNA was extracted from cerebellum using RNAiso plus (Takara, 9109). To eliminate DNA contamination, a Turbo DNA enzyme (Invitrogen, BL698A) was employed. The concentration of total RNA was quantified using the Qubit RNA HS assay kit (Thermo Fisher Scientific, Q32852). Three sets of samples were collected as biological replicates.

### m^6^A-RIP-seq

The MeRIP sequencing process was adapted from a low-input m^6^A-seq protocol. Total RNA was fragmented into approximately 200 nt. For m^6^A input, 10 ng fragmented RNA was used, while the remaining RNA was allocated to m^6^A-seq. Anti-m^6^A antibody-coated magnetic beads were washed with IP buffer before binding the RNA, which was then eluted with *N*6-methyladenosine. The eluted RNA was purified using RNeasy MiniElute spin columns and prepared for sequencing with the SMARTer Stranded Total RNA-Seq Kit v3-Pico Input Mammalian (TaKaRa, 634488). PCR cycles differed between input RNA (14 cycles) and m^6^A RNA (16 cycles). Sequencing was performed on an Illumina NovaSeq with a 150 bp paired-end read length.

### ATAC-seq and CUT&Tag-seq

The frozen tissues were homogenized in a pre-cooled buffer and then filtered to obtain the filtrate. Nuclei in the filtrate were counted using an automated counter. ATAC-seq and CUT&Tag assays were carried out using the ATAC-Seq Kit (Vazyme, TD711-02) and the CUT&Tag Assay Kit (Vazyme, TD903-01) by following the manufacturer’s instructions respectively. Sequencing was conducted on a NovaSeq6000 platform using PE 150 bp read length.

### RNA m^6^A dot blot assay

To conduct the m^6^A dot blot assay, the indicated amount of total RNA was denatured at 95 °C for 5 min, followed by chilling on ice. The RNA was spotted on an Amersham Hybond N^+^ membrane and crosslinked twice with UV. The membrane was blocked with 5% skim milk for 1 h, which was then incubated with the anti-m^6^A antibody (CST, 56593S) overnight at 4 °C or stained with methylene blue (MB) as a reference for normalization. After washing and incubation with secondary antibodies, RNA signals were detected using an enhanced chemiluminescence kit and scanned. The imprints were then analyzed using Image J software.

### m^6^A-seq data analysis

Model -based Analysis for ChIP-Seq(MACS2) (v2.2.7) was used to identify m^6^A peaks in each m^6^A-IP sample with the corresponding input sample as a control. Annotated peak files were generated using Genomic Features and ChIP seeker. Gene ontology(GO) enrichment analysis, Kyoto encyclopedia of genes and genomes(KEGG) enrichment analysis and Reactome enrichment analysis used ClusterProfiler and ggplot2. Read the coverage of m^6^A peaks visualized using Integrative Genomics Viewer(IGV) software (v2.4.15). We filtered the differentially expressed genes based on *P* value < 0.05.

### ATAC-seq data processing

In brief, trim_galore and trimmomatic were used for quality control of low-quality data, bowtie2 was used for comparison, samtools was used to sort the comparison data and remove mitochondrial sequences, sambamba was used to remove repeat sequences, and MACS2 was used to perform peak calling. We filtered the differentially expressed genes based on *P* value < 0.05.

### CUT&Tag data processing

In brief, trim_galore and trimmomatic are used for quality control of low-quality data, bowtie2 is used for comparison, samtools is used to sort comparison data, and SEACR is used for peak calling. We filtered the differentially expressed genes based on *P* value < 0.05.

### Statistical analysis

Statistical analyses were performed using GraphPad Prism v9.1.1 software. Unpaired *t* test was used for statistical testing, and the error bar was shown based on SD (unless stated otherwise). *P* value < 0.05 was considered statistically significant.

## Results

### *Fto* deletion induced abnormalities of fetal cerebellar development.

**Fig. 1:**
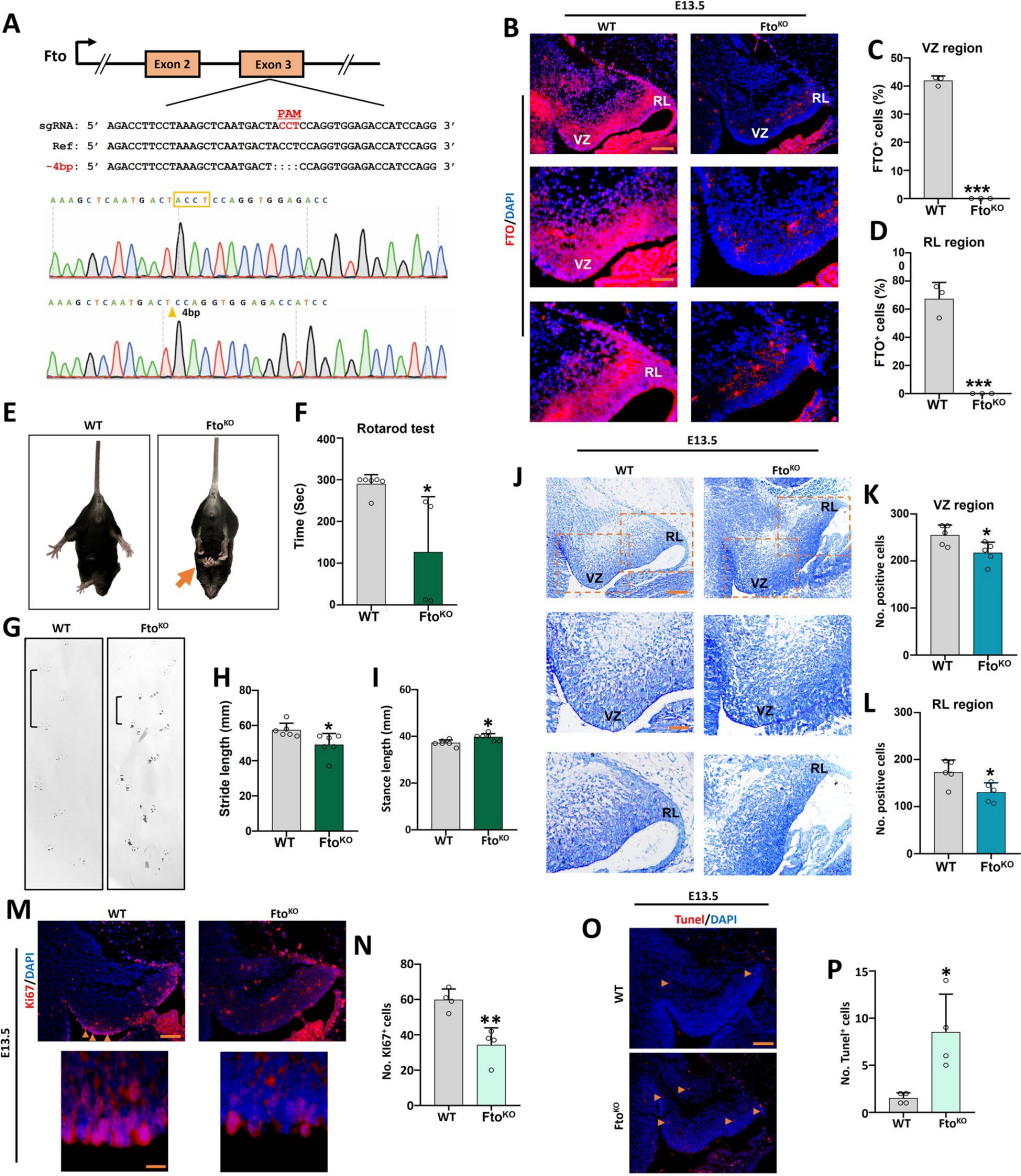
Characterization and phenotype of the *Fto* knockout mice in the cerebellum.** A**. Schematic strategy for constructing the *Fto*^*KO*^ mouse model and the result of Sanger sequencing verification. **B–D** Immunofluorescence analysis of E13.5 cerebellar paraffin sections using an FTO antibody (red) and DAPI (blue) in WT and *Fto*^*KO*^ mice (**B**). The right panels show the percentage of FTO positive cells in the VZ region (**C**) and RL region (**D**), n = 3. Scale bar. 100 μm for top panels; 50 μm for middle and bottom panels. **E** Hindlimb clasping phenotype of 8-week-old *Fto*^*KO*^ mice during tail suspension. The orange arrowheads indicate the clasped feet. **F** Rotarod test analysis of WT and *Fto*^*KO*^ mice. n = 6. **G–I**. Footprint pattern analysis showing the blue ink trace of WT and *Fto*^*KO*^ mice. Statistical analysis of the distance of stride length (**H**), and stance length (**I**) of WT and *Fto*^*KO*^ mice, n = 6. **J–L**. Nissl-stained sagittal cerebellar sections from WT and *Fto*^*KO*^ mice at E13.5 (**J**). Scale bar, 100 μm for top panels; 50 μm for middle and bottom panels. The right panel shows the positive neuron numbers in the VZ region (**K**) and RL region (**L**) of the cerebellum at E13.5, n = 5. **M**, **N**. Immunofluorescence analysis of E13.5 cerebellar paraffin sections using a Ki67 antibody (red) and DAPI (blue). The orange arrowheads indicate Ki67 positive cells. The right panels show the numbers of Ki67 positive cells in WT and *Fto*^*KO*^ mice, n = 4. Scale bar, 100 μm. **O**, **P**. TUNEL analysis of E13.5 cerebellar paraffin sections. The orange arrowheads indicate apoptotic body. The right panels show the numbers of TUNEL positive cells in WT and *Fto*^*KO*^ mice, n = 4. Scale bar, 100 μm. *, *P* < 0.05; **, *P* < 0.01; ***, *P* < 0.001. Student *t* test. All data are presented as the means ± SD. E13.5, embryonic day 13.5; VZ, ventricular zone; RL, rhombic lip; SD, standard deviation

To investigate the role of *Fto* in fetal cerebellar development, we generated a Fto knockout (*Fto*^*KO*^) mouse model using CRISPR/Cas9 to delete 4 bp in the exon 3 of *Fto* (Fig. 1A). We observed a significant reduction in protein expression of FTO in the cerebellum of *Fto*^*KO*^ mice at the stage of E13.5 (Supplementary Fig. 1A). Immunofluorescence analysis of cerebellar tissues at an early development stage of E13.5 and P3 revealed that the FTO positive cells were notably reduced (Fig. 1B–D and Supplementary Fig. 1B, C). By eight weeks, the *Fto*^*KO*^ mice had developed severe tremors with tightly curled legs and an abnormal hindlimb clasping reflex after being suspended by their tails, demonstrating typical features of cerebellar ataxia (Fig. 1E). Meanwhile, the rotarod test demonstrated that *Fto*^*KO*^ mice had far less effective motor coordination (Fig. 1F). Footprint pattern analysis showed that the *Fto*^*KO*^ mice had an abnormal gait characterized by reduced movement distance and increased stance length (Fig. 1G–I). Immunofluorescence results indicated that *Fto* deficiency reduced the number of Pax6 and Calbindin positive cells (Supplementary Fig. 1D-G). Nissl staining indicated that *Fto*^*KO*^ decreased the overall cerebellar positive cell numbers in the ventricular zone (VZ) and rhombic lip (RL) at E13.5 and the external granule cell layer (EGL) thickness at P3 (Fig. 1J-L and Supplementary Fig. 1H, I). Furthermore, immunofluorescence revealed that *Fto* deletion at E13.5 and P3 reduced proliferation while increasing apoptosis (Fig. 1M-P and Supplementary Fig. 1 J-M).

### *Fto* deletion affected self-renewal and differentiation in the fetal cerebellum.

**Fig. 2:**
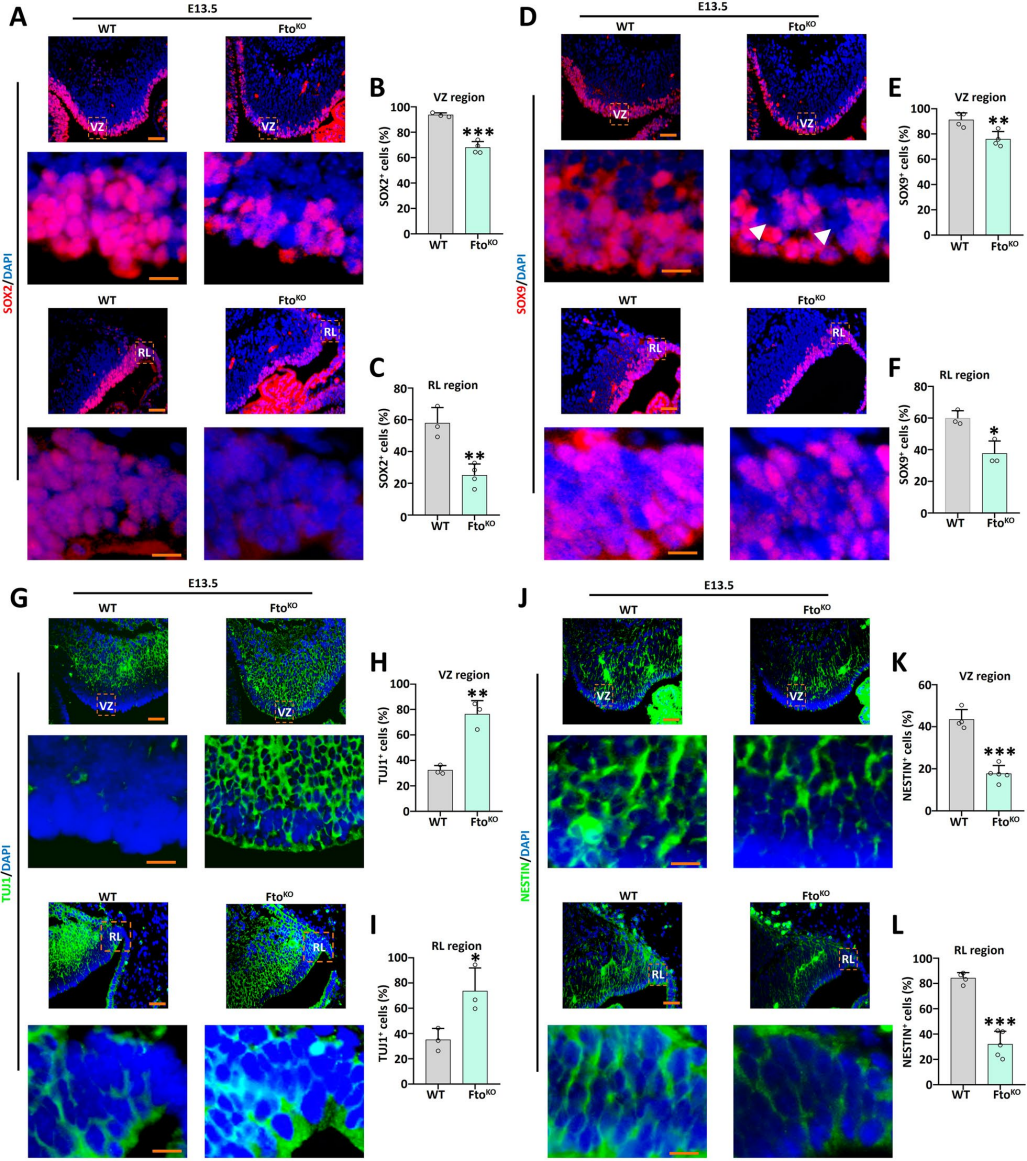
*Fto* knockout affected the development of fetal cerebellum.** A**–**C.** Immunofluorescence analysis of E13.5 cerebellar paraffin sections using a SOX2 antibody (red) and DAPI (blue). The right panels show the percentage of SOX2 positive cells in VZ region (**B**) and RL region (**C**), n = 3. Scale bar, 50 μm. **D**–**F**. Immunofluorescence analysis of E13.5 cerebellar paraffin sections using a SOX9 antibody (red) and DAPI (blue). The right panels show the percentage of SOX9 positive cells in VZ region (**E**) and RL region (**F**), n = 3. Scale bar, 50 μm. **G**–**I**. Immunofluorescence analysis of E13.5 cerebellar paraffin sections using a TUJ1 antibody (green) and DAPI (blue). The right panels show the percentage of TUJ1 positive cells in VZ region (**H**) and RL region (**I**), n = 3. Scale bar, 50 μm. **J**–**L**. Immunofluorescence analysis of E13.5 cerebellar paraffin sections using a NESTIN antibody (green) and DAPI (blue). The right panels show the percentage of NESTIN positive cells in VZ region (**K**) and RL region (**L**), n = 3. Scale bar, 50 μm. *, *P* < 0.05; **, *P* < 0.01; ***, *P* < 0.001. Student *t* test. All data are presented as the means ± SD. E13.5, embryonic day 13.5; VZ, ventricular zone; RL, rhombic lip; SD, standard deviation

To confirm whether *Fto* affects cerebellar development, immunofluorescence examination of cerebellar tissues at the stage of E13.5 revealed that *Fto*^*KO*^ decreased the neural self-renewal gene *Sox2* and *Sox9* positive cells in the VZ and RL regions (Fig. 2A–F). At the P3 stage, the neural self-renewal genes *Sox2*, *Sox9* and *Pax6* expression was inhibited in the internal granule cell layer (IGL) region (Supplementary Fig. 2A-F). Moreover, the immunofluorescence method found that *Fto*^*KO*^ elevated the neural differentiation gene *Tuj1* expression in the VZ and RL regions (Fig. 2G-I), the similar pattern was discovered at the P3 stage in the external granule cell layer (EGL) region (Supplementary Fig. 2G, H). Meanwhile, immunofluorescence results revealed that *Fto*^*KO*^ decreased the neural stem cells marker *Nestin* expression at E13.5 and the neuron marker *Map2* expression at P3 (Fig. 2J–L, Supplementary Fig. 2I, J).

### m^6^A RIP-seq for ***Fto***^***KO***^ fetal cerebellar tissues and ***Fto*** deletion affected the levels of H4K16ac.

**Fig. 3:**
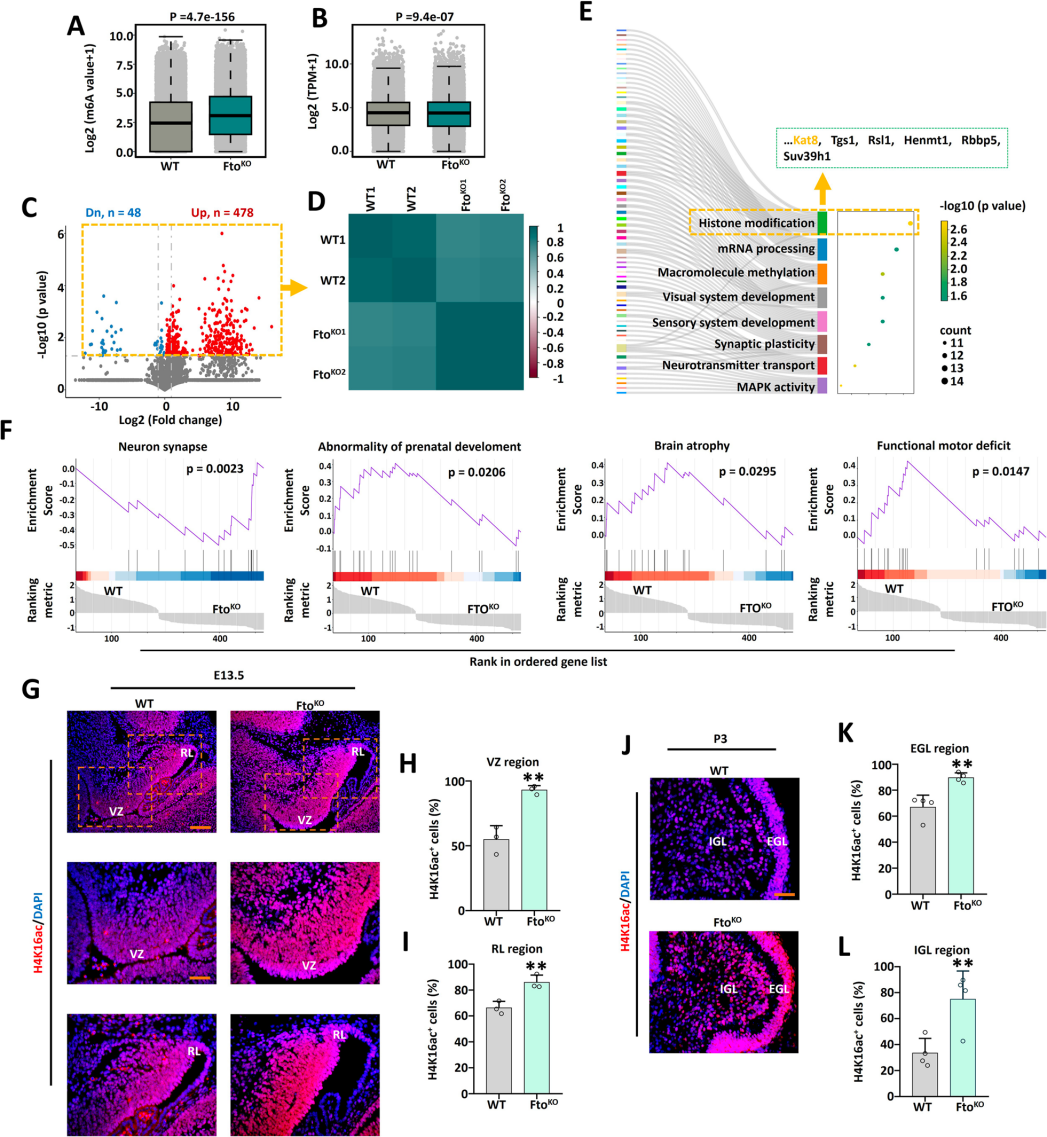
*Fto* deficiency induced high levels of m^6^A and abnormal histone modification. **A**, **B**. The m^6^A modification and mRNA levels were compared in WT and *Fto*^*KO*^ mice at E13.5. Data represent the mean of two independent experiments, each containing two biological replicates. We used Wilcoxon Rank-Sum Test in data analysis. **C**. Volcano plots showing m^6^A modificatory differential genes in WT and *Fto*^*KO*^ mice at E13.5. There were 48 down-regulated m^6^A modificationgenes, 478 up-regulated m^6^A modification genes, standard with *P* < 0.05. We used Wilcoxon Rank-Sum Test in DATA Analysis. **D**. The correlation analysis was carried out in WT and *Fto*^*KO*^ mice at E13.5. **E**. GO pathway enrichment analyses were carried out for m^6^A modificatory differential genes. Histone acetyltransferase Kat8 from histone modification pathway was found. **F**. The GSEA analysis showing the association of m^6^A differential genes in neuron synapse, abnormality of prenatal development, brain atrophy and functional motor deficit. **G**–**I**. H4K16ac (red) and DAPI (blue) immunofluorescent staining of WT and *Fto*^*KO*^ mice at E13.5 (**G**). Scale bar, 100 μm for top panels; 50 μm for middle and bottom panels. The right panel shows the percentage of H4K16ac positive cells in the VZ region (**H**) and RL region (**I**), n = 3. **J**–**L**. H4K16ac (red) and DAPI (blue) immunofluorescent staining of WT and *Fto*^*KO*^ mice at P3 (**J**). Scale bar, 50 μm. The right panel shows the percentage of H4K16ac positive cells in the EGL (**K**) and IGL (**L**), n = 3. *, *P* < 0.05; **, *P* < 0.01. Student *t* test. All data are presented as the means ± SD. E13.5, embryonic day 13.5; P3, postnatal day 3; VZ, ventricular zone; RL, rhombic lip; EGL, external granule cell layer; IGL, internal granule cell layer; GO, Gene ontology. GSEA, gene set enrichment analysis. SD, standard deviation

To investigate the regulatory mechanism of *Fto* for fetal cerebellar function, m^6^A RIP-seq was employed to test the cerebellum following *Fto* loss. The total m^6^A levels increased dramatically after *Fto*^*KO*^, and while the mRNA levels were elevated slightly after *Fto* deletion (Fig. 3A–B). The difference analysis found that 478 up-regulated and 48 down-regulated m^6^A modification genes (Fig. 3C). Correlation analysis of the m^6^A difference genes revealed high similarity within the same group, but significant diversity between the WT and *Fto*^*KO*^ groups (Fig. 3D). The GO analysis was performed using m^6^A difference genes, which were mainly enriched in histone modification, sensory system development and neurotransmitter transport signaling pathways (Fig. 3E). And then the gene set enrichment analysis (GSEA) method was carried out utilizing m^6^A difference genes, the results indicated that the difference genes were related to neuron synapse, abnormality of prenatal development, brain atrophy, functional motor deficit, ESR1 targets and cell migration (Fig. 3F, Supplementary Fig. 3A). In addition, to determine if *Fto*^*KO*^ caused the fetal cerebellar function loss by affecting histone modification, the immunofluorescence method was utilized to assess the *Fto*^*KO*^ fetal cerebellar tissues. The results indicated that the H4K16ac and H3K27ac positive cells were raised in *Fto*^*KO*^ fetal cerebellar tissues at E13.5 in the VZ and RL regions (Fig. 3G-I and Supplementary Fig. 3B-D). At P3 stage, the number of H4K16ac and H3K27ac positive cells were elevated in *Fto*^*KO*^ fetal cerebellar tissues in the EGL and IGL regions (Fig. 3J–L and Supplementary Fig. 3E-G).

### ***Fto***^***KO***^ affected the transcriptional level through regulating H4K16ac.

**Fig. 4:**
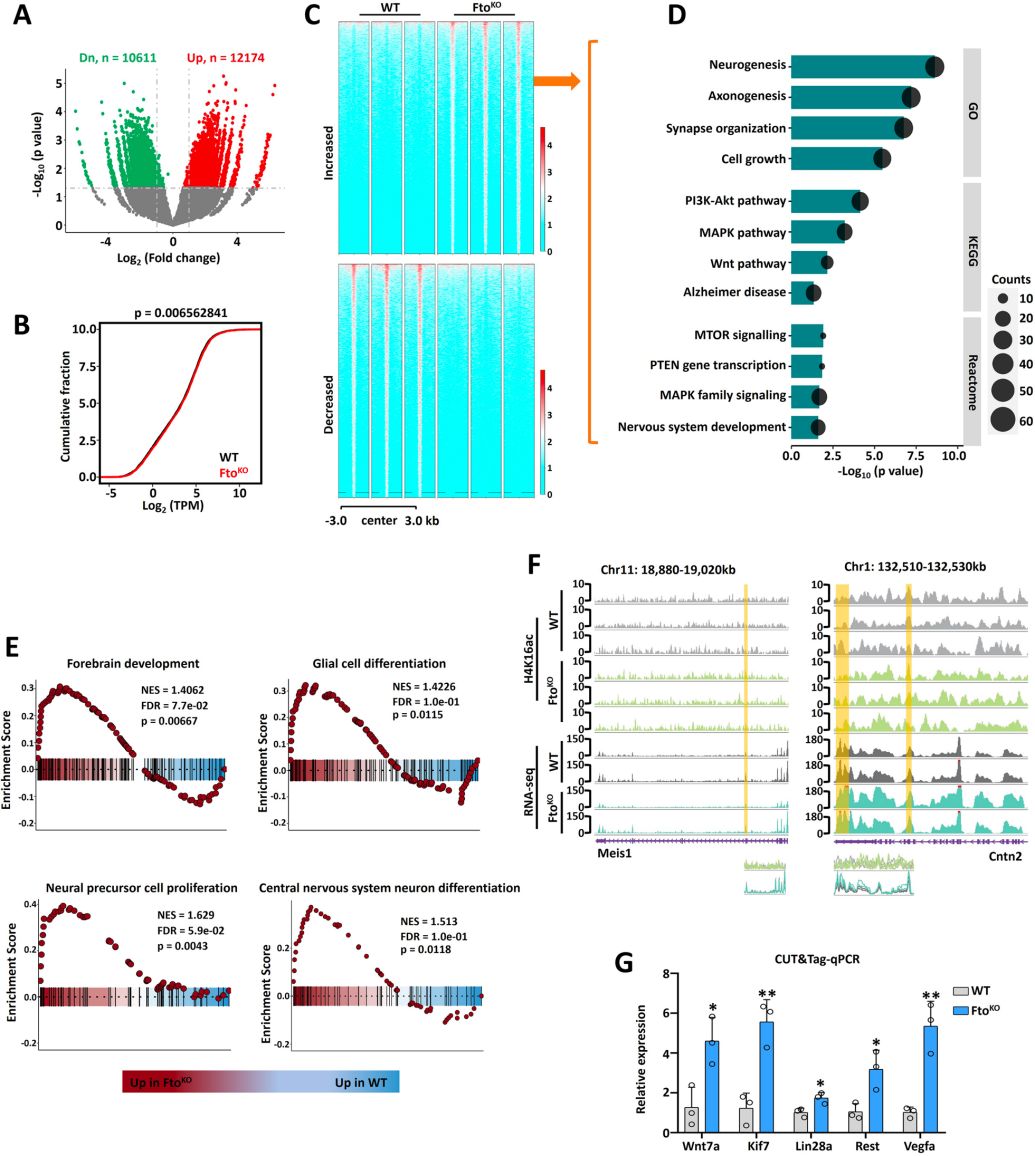
*Fto* affected gene transcription by regulating H4K16ac in cerebellar development. **A**. Volcano plots showing differential peaks from CUT&Tag-seq for H4K16ac in WT and *Fto*^*KO*^ mouse cerebellums at E13.5. Significantly upregulated and downregulated genes are marked as red and green dots, respectively. The cut-off standard is *P* value less than 0.05. We used Wilcoxon Rank-Sum Test in data analysis. There were 10,611 down-regulated m^6^A modification genes, 12,174 up-regulated m^6^A modification genes, standard with *P* < 0.05. Data represent the mean of two independent experiments, n = 3. **B**. The transcription level analysis was carried out for statistically differential peaks derived genes. We used Wilcoxon Rank-Sum Test in data analysis. **C**. The statistically differential peaks from CUT&Tag-seq for H4K16ac were divided into increased and decreased groups. **D**. GO-KEGG-Reactome analyses were used with the genes from statistically different sites from the increased group. The differentially expressed genes were related to the signaling pathways of nervous system development such as neurogenesis and axonogenesis. **E**. The GSEA analysis showing the association of statistically differential peaks derived genes in forebrain development, glia cell differentiation, neural precursor cell proliferation and central nervous system neuron differentiation. **F**. IGV demonstrated the distinct enrichment in H4K16ac and mRNA of the fetal cerebellar function of regulatory genes (*Meis1* and *Cntn2*). **G**. CUT&Tag-qPCR showing the level of H4K16ac modification on the neurogenesis regulatory genes in WT and *Fto*^*KO*^ mice at E13.5 (*Wnt7a*, *Kif7*, *Lin28a*, *Rest*, *Vegfa*). n = 3. GO, Gene ontology. KEGG, Kyoto encyclopedia of genes and genomes. GSEA, gene set enrichment analysis. IGV, Integrative Genomics Viewer

In order to confirm whether *Fto*^*KO*^ inhibits fetal cerebellar function by affecting H4K16ac modification, the CUT&Tag-seq was performed on *Fto*^*KO*^ fetal cerebellar tissues. We identified 10,611 down-regulated sites and 12,174 up-regulated sites after *Fto*^*KO*^ in the promoter and exon regions (Fig. 4A). The transcriptional level at the statistically differential sites was promoted (Fig. 4B). The histone H4K16ac-enriched statistically differential sites were divided into increased and decreased groups when *Fto* was deleted (Fig. 4C), GO-KEGG-Reactome analyses were used with the genes from statistically differential sites from the increased group, and the findings exhibited that different genes were associated with neurogenesis, axonogenesis, MAPK, PI3K-Akt, MTOR and nervous system development signaling pathways (Fig. 4D). GSEA analysis of these statistically different genes revealed associations with forebrain development, glia cell differentiation, neural precursor cell proliferation and central nervous system neuron differentiation signaling pathways and physiological functional regulatory signaling pathways (G2/M phase transition, telomere maintenance, phagocytosis, and PI3K-Akt) in Fig. 4E and Supplementary Fig. 4A. IGV showed distinct enrichment in H4K16ac and mRNA in the fetal cerebellar functional regulatory genes *Meis1* and *Cntn2* (Fig. 4F). Additionally, according to the neurogenesis related genes from GO analysis, we employed CUT&Tag-qPCR method to evaluate H4K16ac occupancy at the promoter regions of target genes, the results found that *FTO*^*KO*^ affected neural function (*Wnt7a*, *Kif7*, *Lin28a*, *Rest* and *Vegfa*), differentiation (*Tuj1*) and self-renewal (*Nestin* and *Sox9*) in Fig. 4G and Supplementary Fig. 4B.

### ***Fto***^***KO***^ influenced chromatin accessibility through regulating H4K16ac.

**Fig. 5:**
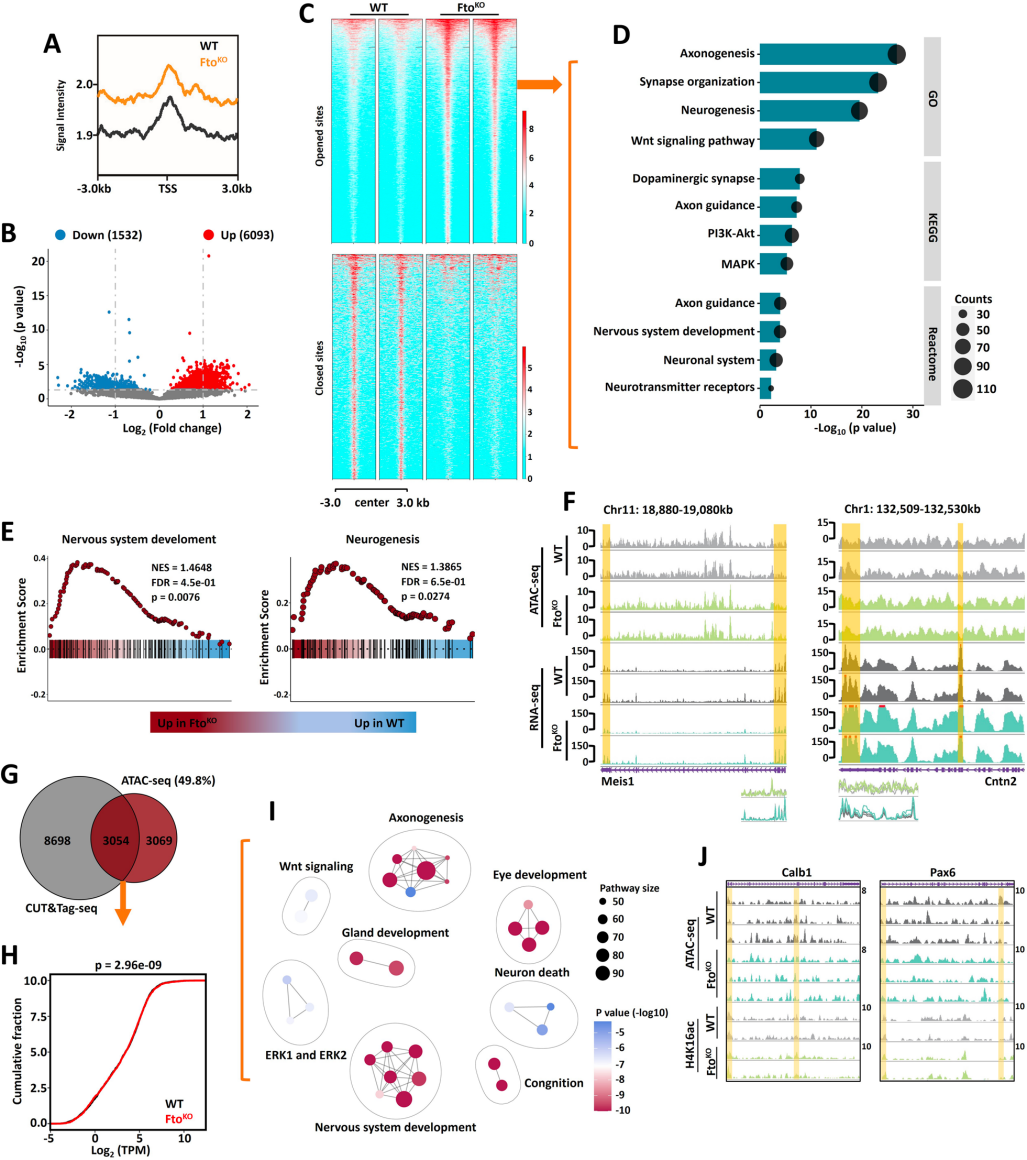
*Fto*^*KO*^ influenced the chromatin accessibility via regulating H4K16ac. **A**. Aggregate total ATAC-seq enrichment plots for WT and *Fto*^*KO*^ mice signals at transcriptional start site. We used Wilcoxon Rank-Sum Test in data analysis. **B**. Volcano plots showing differential peaks from ATAC-seq in WT and *Fto*^*KO*^ mice at E13.5. The cut-off standard is *p* value less than 0.05. We used Wilcoxon Rank-Sum Test in data analysis. n = 2. **C**. The statistically differential peaks from ATAC-seq were divided into opened sites and closed sites groups. **D**. GO-KEGG-Reactome analyses were carried out for analyzing opened sites groups statistically differential peaks derived genes. **E**. The GSEA analysis showing the association of statistically differential peaks derived genes in nervous system development and neurogenesis. **F**. IGV demonstrated the distinct enrichment in chromatin and mRNA of the fetal cerebellar function of regulatory genes (*Meis1* and *Cntn2*). **G**. The Venn diagram analysis was carried out for H4K16ac and ATAC-seq common genes (49.8%) with statistic difference after *Fto*^*KO*^*.* **H**. The transcription level analysis was promoted for common genes. **I**. GO analysis indicates that common genes were associated with axonogenesis, neuron death and nervous system development signaling pathways. **J**. IGV demonstrated that colocalization peaks from H4K16ac and chromatin in the fetal cerebellar functional regulatory genes (*Calb1* and *Pax6*). GO, Gene ontology. KEGG, Kyoto encyclopedia of genes and genomes. GSEA, gene set enrichment analysis. IGV, Integrative Genomics Viewer.

The histone modification is linked with chromatin stability [26]. We employed ATAC-seq to estimate whether the *Fto* regulated gene transcription process is associated with chromatin accessibility alterations. The distribution of total sites was promoted at the TSS region when *Fto* was deleted (Fig. 5A). We identified 1532 down-regulated sites and 6093 up-regulated sites after *Fto*^*KO*^ from ATAC-seq (Fig. 5B). The chromatin-enriched statistically differential sites were separated into increased and decreased groups, and the enriched sites tended to undergo transcription enhancement when *Fto* was deleted (Fig. 5C). GO-KEGG-Reactome analyses were used with the genes from statistically differential sites in increased group, and the findings exhibited that different genes were associated with axonogenesis, synapse organization, neurogenesis, axon guidance and nervous system development signaling pathways (Fig. 5D). GSEA analysis of these statistically different genes was associated with nervous system development, neurogenesis, distal axon, neuronal cell body, behavior and double-stranded DNA binding signaling pathways in Fig. 5E and Supplementary Fig. 5A. IGV demonstrated distinct enrichment in chromatin and mRNA in the fetal cerebellar function of regulatory genes *Meis1* and *Cntn2* (Fig. 5F). In order to confirm the link between H4K16ac and chromatin, a Venn diagram analysis was carried out on the statistically different genes after *Fto*^*KO*^. The results found that there were 3054 common genes between H4K16ac and chromatin (Fig. 5G), and the transcriptional level was elevated (Fig. 5H). GO analysis indicates that common genes were associated with axonogenesis, neuron death and nervous system development signaling pathways (Fig. 5I). Meanwhile, IGV detected colocalization peaks from H4K16ac and chromatin in the fetal cerebellar functional regulatory genes *Calb1* and *Pax6* (Fig. 5J). IGV demonstrated distinct enrichment in H4K16ac and chromatin in the fetal cerebellar function of regulatory genes *Atoh1* promoter regions (Supplementary Fig. 5B). Meanwhile, in order to confirm that whether *Fto* loss affect the mRNA level of *Atoh1*, real-time PCR assay was carried out. The results found that *Fto* deletion decreased the transcription level of *Atoh1* in the cerebellar tissue at E13.5 (Supplementary Fig. 5C). Meantime, we employed the immunofluorescence method to test. The results indicated that *Fto* deletion decreased the number of ATOH1 positive cells during cerebellar development at E13.5 (Supplementary Fig. 5D, E).

### Fto/Kat8/Igf2bp3 regulatory axis influenced the cerebellar development.

**Fig. 6:**
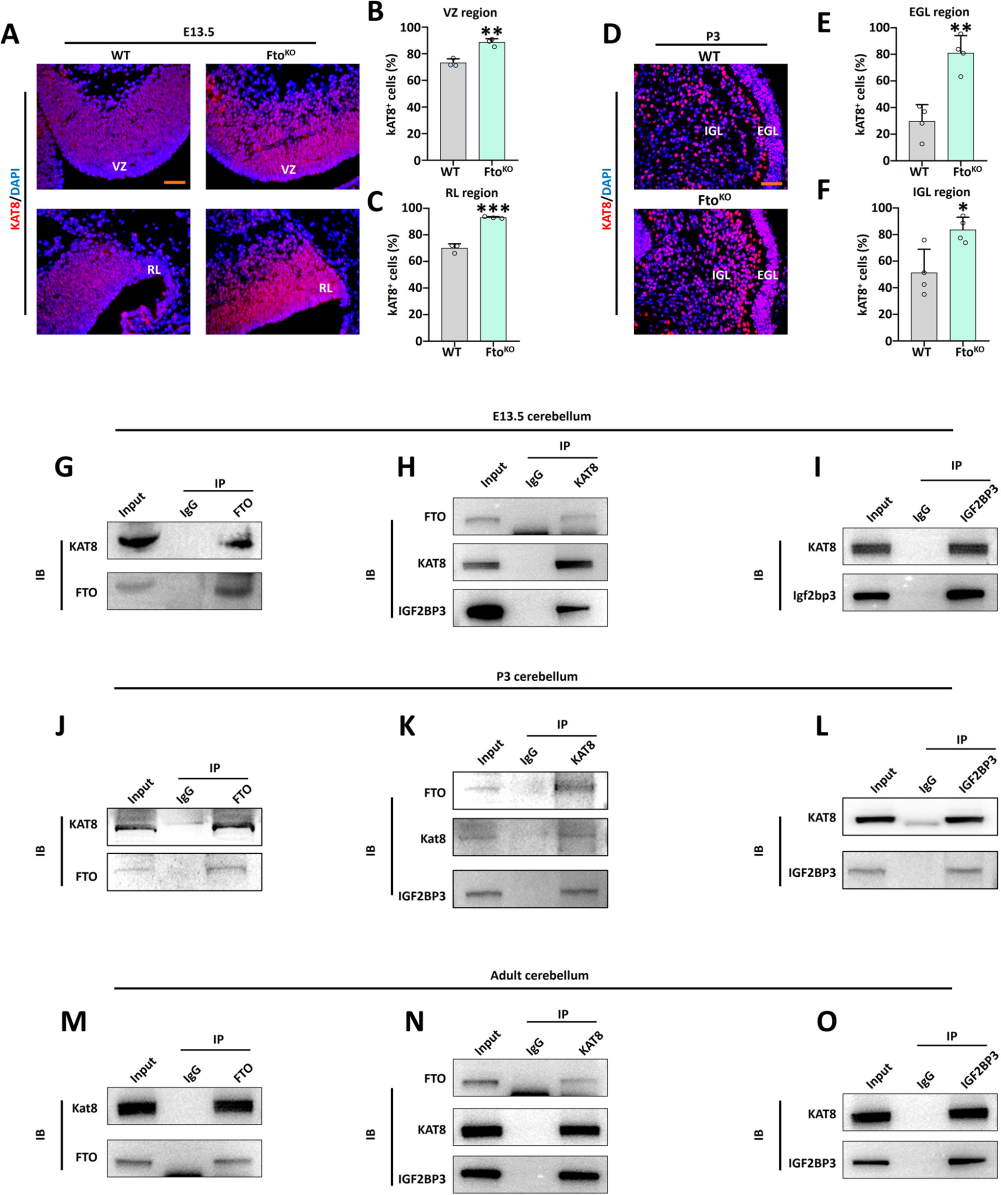
*Fto* recruited acetyltransferase KAT8 to affect fetal cerebellar development.** A**–**C**. KAT8 (red) and DAPI (blue) immunofluorescent staining in the VZ and RL regions of WT and *Fto*^*KO*^ mice at E13.5 (**A**). Scale bar, 50 μm. The right panels show the percentage of KAT8 positive cells in the VZ and RL regions of WT and *Fto*^*KO*^ mice at E13.5 (**B**, **C**), n = 3. **D**–**F**. KAT8 (red) and DAPI (blue) immunofluorescent staining in the EGL and IGL regions of WT and *Fto*^*KO*^ mice at P3 (**D**). Scale bar, 50 μm. The right panel shows the percentage of KAT8 positive cells in the EGL and IGL regions of WT and *Fto*^*KO*^ mice at P3 (**E**, **F**), n = 3. **G**–**O**. Co-immunoprecipitation experiments were conducted using mouse cerebellum lysates at different developmental stages. IgG was used as a negative control. FTO was co-immunoprecipitated from E13.5 mouse cerebellum lysates, followed by immunoblot analysis of KAT8 and FTO (**G**). KAT8 was co-immunoprecipitated from the same E13.5 mouse cerebellum lysates, and immunoblot analysis was performed for FTO, KAT8, and IGF2BP3 (**H**). IGF2BP3 was co-immunoprecipitated from E13.5 mouse cerebellum lysates, followed by immunoblot analysis of KAT8 and IGF2BP3 (**I**). The same co-immunoprecipitation and immunoblot procedures were repeated for P3 mouse cerebellum lysates, involving FTO, KAT8, and IGF2BP3 (**J**–**L**). The same co-immunoprecipitation and immunoblot procedures were repeated for adult mouse cerebellum lysates, involving FTO, KAT8, and IGF2BP3 (**M**–**O**). *, *P* < 0.05; **, *P* < 0.01. Student *t* test. All data are presented as the means ± SD. E13.5, embryonic day 13.5; P3, postnatal day 3; VZ, ventricular zone; RL, rhombic lip; IGL, internal granule cell layer; EGL, external granule cell layer. IP, immunoprecipitation. IB, immunoblotting. SD, standard deviation

**Fig. 7:**
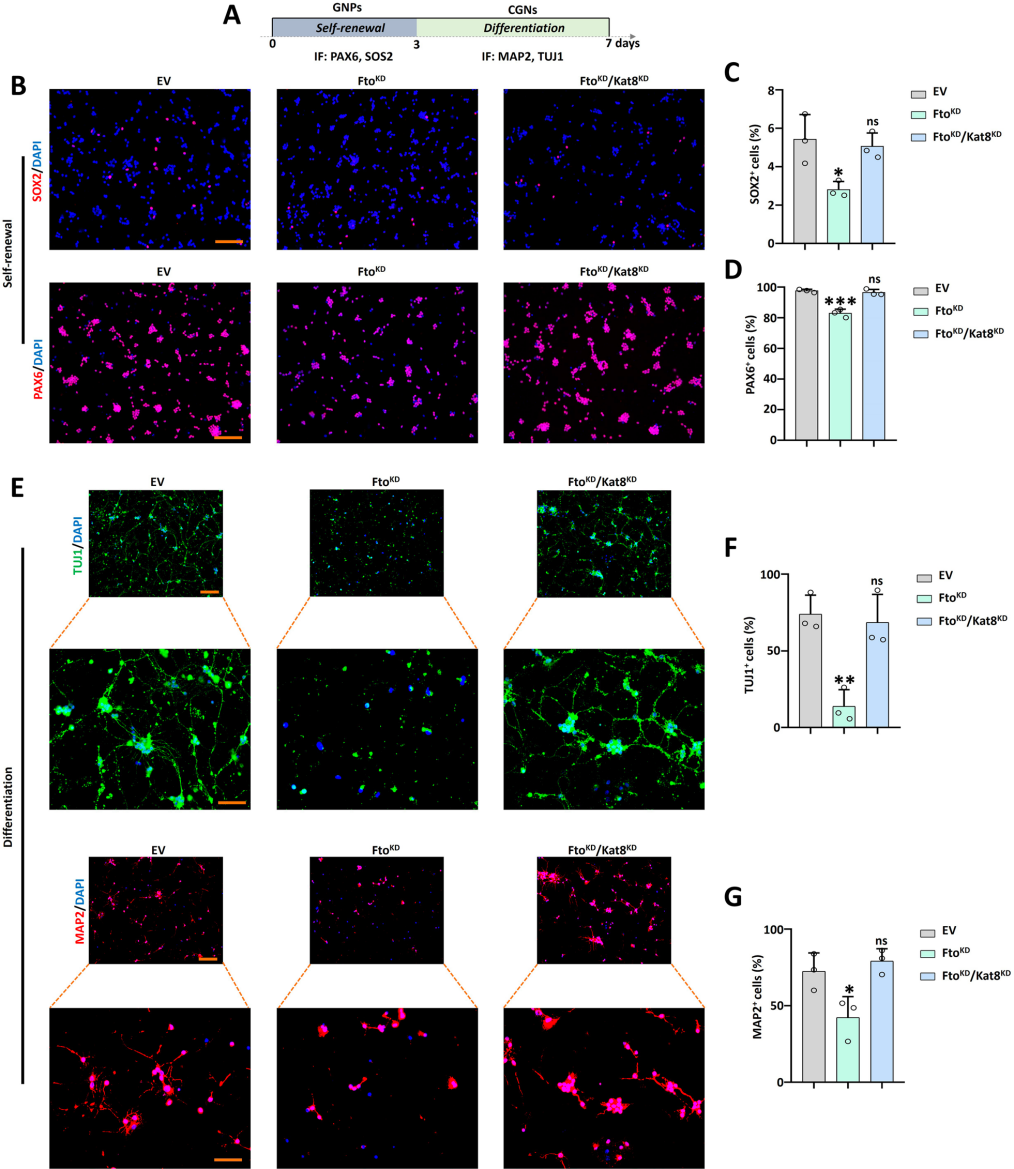
*Kat8*^*KD*^ restored neural self-renewal and differentiation in *Fto*^*KD*^ granule neuron precursors (GNPs). **A**. Diagram illustrating the workflow of self-renewal and differentiation in primary GNPs in vitro. **B**–**D**. SOX2 (red), PAX6 (red) and DAPI (blue) immunofluorescent staining of EV, *Fto*^*KD*^, and *Fto*^*KD*^/*Kat8*^*KD*^ (**B**). Scale bar, 100 μm. The right panel shows the percentage of SOX2 positive cells (**C**) and Pax6 positive cells (**D**) in three groups, n = 3. **E**–**G** TUJ1 (green), MAP2 (red) and DAPI (blue) immunofluorescent staining of EV, *Fto*^*KD*^, and *Fto*^*KD*^/*Kat8*^*KD*^ (**E**). Scale bar, 100 μm for top panels; 50 μm for bottom panels. The right panel shows the percentage of TUJ1 positive cells (**F**) and Map2 positive cells (**G**) in three groups. n = 3. *, *P* < 0.05; **, *P* < 0.01; ***, *P* < 0.001. Student *t* test. All *P*-values are compared with the EV group. All data are presented as the means ± SD. EV, empty vector. KD, knockdown. GNPs, granule neuron precursors. CGNs, cerebellar granule neurons. SD, standard deviation

To examine the regulatory link between FTO and KAT8, the immunofluorescence (IF) was performed on the *Fto*^*KO*^ cerebellar tissues. We discovered that the number of KAT8 positive cells increased in the VZ and RL regions at E13.5 (Fig. 6A–C), the same situation was found at the P3 stage in the EGL and IGL regions (Fig. 6D–F). Collected cerebellar tissues at E13.5, Co-IP verified that FTO could combine with KAT8. Meanwhile, KAT8 could combine to FTO and the m^6^A reader protein IGF2BP3 (Fig. 6G, H). In reverse, IGF2BP3 has the ability to combine with KAT8 (Fig. 6I). Moreover, similar results were found at the P3 and adult stages (Fig. 6J-O). Meanwhile, KAT8 showed weak binding to other m^6^A readers IGF2BP1 or IGF2BP2. Kat8 was hard to combine with the other m^6^A readers protein Igf2bp1 or Igf2bp2 (Supplementary Fig. 6A). Moreover, *Fto*^*KO*^ reduce the combining capacity between FTO and KAT8 (Supplementary Fig. 6B). In order to confirm the regulatory relationship between FTO and KAT8, the *Fto* knockdown (*Fto*^*KD*^) and *Kat8* knockdown (*Kat8*^*KD*^) granule neuron precursors (GNPs) were generated in the self-renewal and differentiation culture systems (Fig. 7A). The IF results indicated that *Kat8*^*KD*^ restored the self-renewal genes *Sox2 and Pax6* expression in the *Fto*^*KD*^ group (Fig. 7B–D). In addition, when GNPs were differentiated into cerebellar granule neurons (CGNs), the IF results found that *Kat8*^*KD*^ recovered the differentiation markers TUJ1 and MAP2 expression totally in the *Fto*^*KD*^ group (Fig. 7E–G). Although *Kat8* knockdown partially rescues the deficits in GNP proliferation and differentiation, we are considering whether direct manipulation of H4K16ac levels similarly rescues the cerebellar developmental phenotype in Fto-deficient cells. We employed the histone acetyltransferase KAT8 inhibitors MC4171 to test the self-renew markers (SOX2, PAX6) and differentiation marker (MAP2). The result indicated that 10 μM MC4171 could rescue the number of SOX2 positive cells partly in *Fto*^*KD*^ group, 25 μM MC4171 could rescue damaged effect from *Fto*^*KD*^ in SOX2 totally (Supplementary Fig. 7A, B). For the number of PAX6 positive cells, 10 μM and 25 μM MC4171 are all could rescue the influence from *Fto*^*KD*^ completely (Supplementary Fig. 7C, D). Besides, the result indicated that 10 μM MC4171 could rescue the number of MAP2 positive cells partly in *Fto*^*KD*^ group, 25 μM MC4171 could rescue damaged effect from *Fto*^*KD*^ in MAP2 totally (Supplementary Fig. 7E, F). Therefore, we have demonstrated that during the development of the cerebellum, the m^6^A modification mediated by FTO is recognized by IGF2BP3, thereby increasing the expression level of *Kat8* mRNA, and further increasing the modification level of histone H4K16ac by recruiting KAT8. Regulating the modification level of histone H4K16ac can rescue the cerebellar developmental phenotype of *Fto*-deficient cells.

### FTO-mediated m^6^A modification and overexpression of ***Kat8*** affected cerebellar development.

**Fig. 8:**
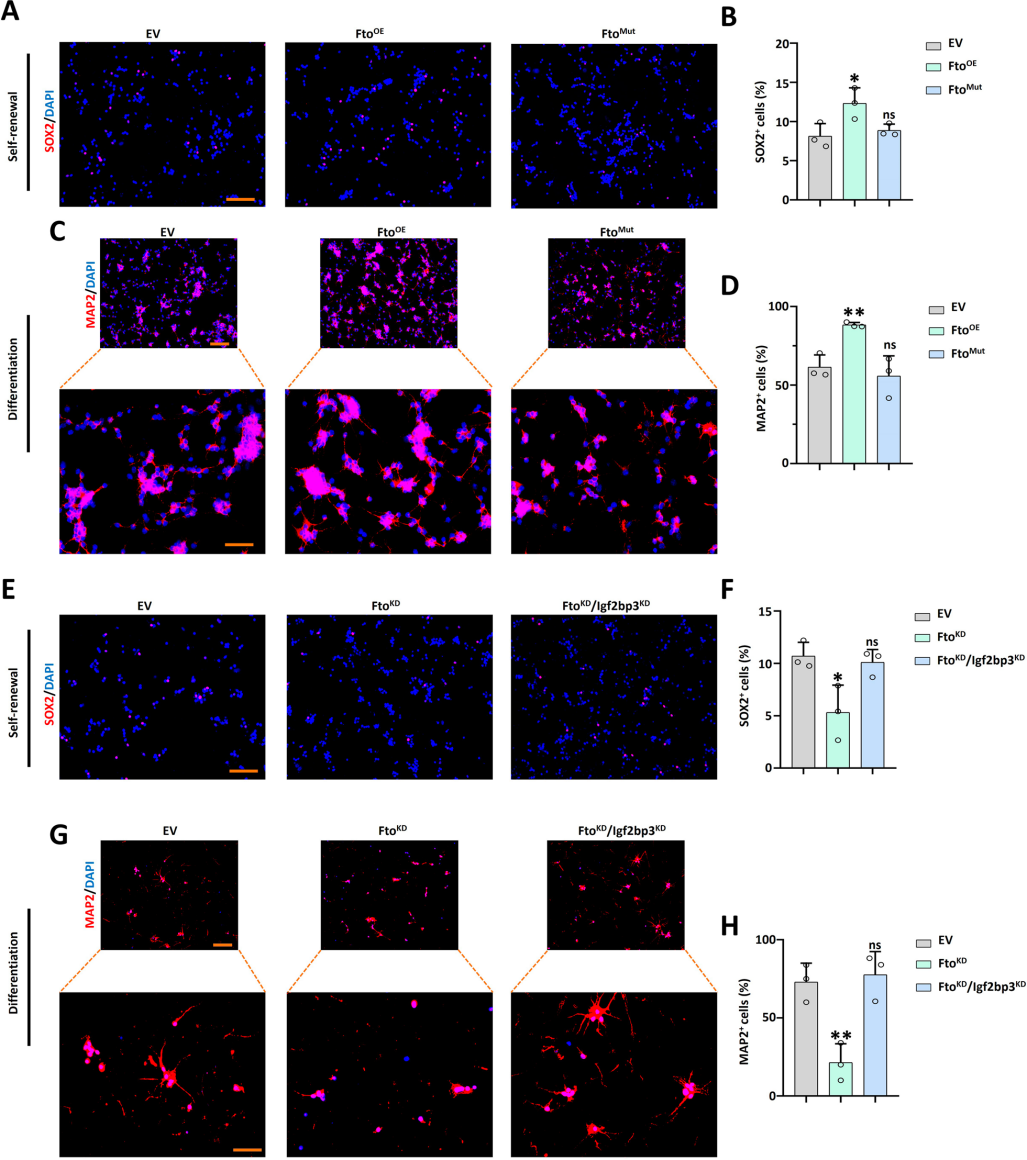
*Fto*-mediated m^6^A modification affected neural self-renewal and differentiation. **A**, **B**. SOX2 (red) and DAPI (blue) immunofluorescent staining of EV, *Fto*^*OE*^ and *Fto*^*Mut*^ groups(**A**). Scale bar, 100 μm. The right panel shows the percentage of SOX2 positive cells (**B**) in three groups, n = 3. **C, D.** MAP2 (red) and DAPI (blue) immunofluorescent staining of EV, *Fto*^*OE*^ and *Fto*^*Mut*^ (**C**). Scale bar, 100 μm for top panels; 50 μm for bottom panels. The right panel shows the percentage of MAP2 positive cells (**D**) in three groups, n = 3. **E, F.** SOX2 (red) and DAPI (blue) immunofluorescent staining of EV, *Fto*^*KD*^ and *Fto*^*KD*^/*Igf2bp3*^*KD*^ groups (**E**). Scale bar, 100 μm. The right panel shows the percentage of SOX2 positive cells (**F**) in three groups, n = 3. **G**, **H**. MAP2 (red) and DAPI (blue) immunofluorescent staining of EV, *Fto*^*KD*^ and *Fto*^*KD*^/*Igf2bp3*^*KD*^ (**G**). Scale bar, 100 μm for top panels; 50 μm for bottom panels. The right panel shows the percentage of MAP2 positive cells (**H**) in three groups, n = 3. *, *P* < 0.05; **,* P* < 0.01; ***, *P* < 0.001. Student *t* test. All *P*-values are compared with the EV group. All data are presented as the means ± SD. EV, empty vector. KD, knockdown. OE, overexpression. MUT, mutant. SD, standard deviation

**Fig. 9:**
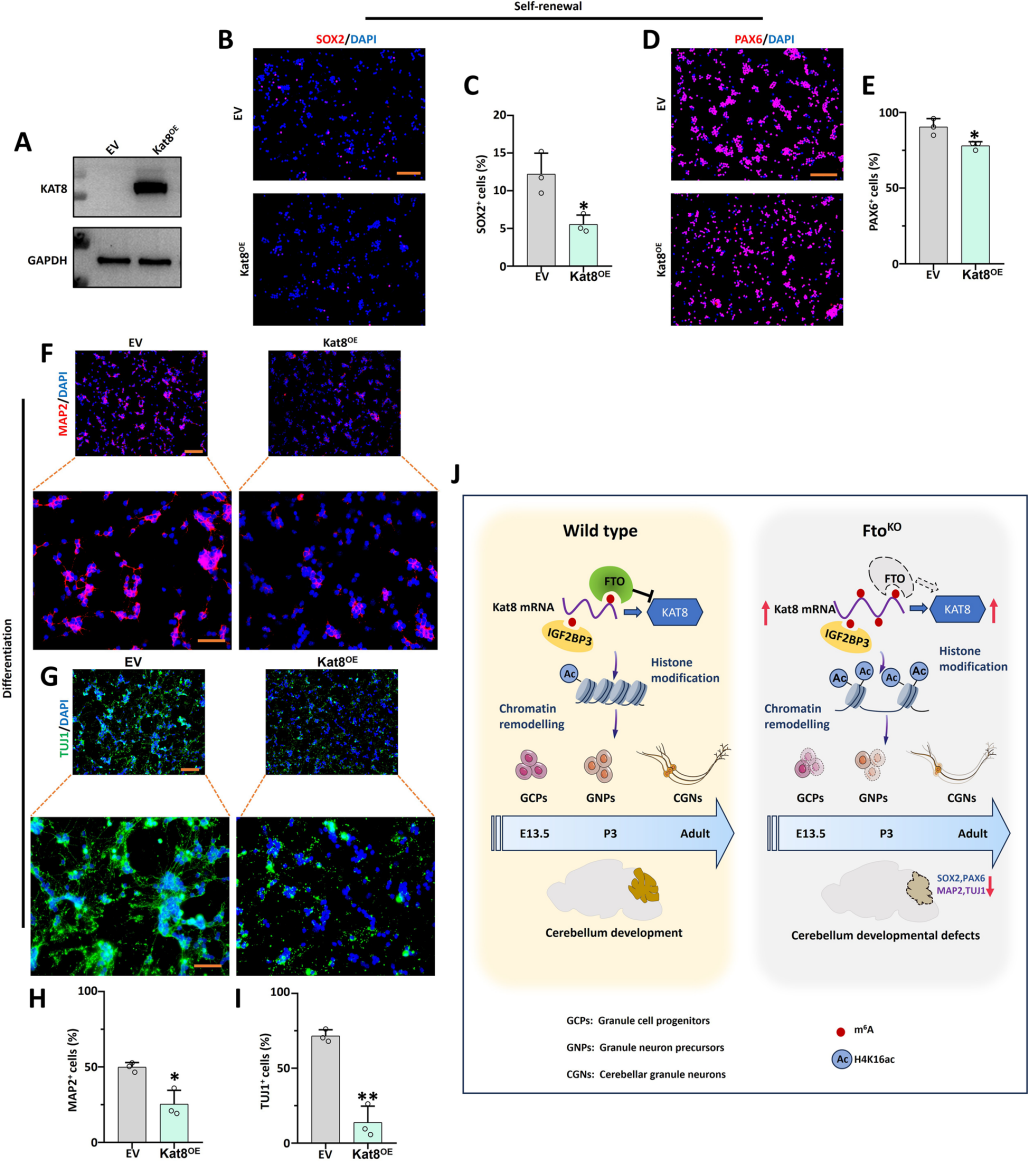
*Kat8* overexpression impacted neural self-renewal and differentiation in GNPs. **A**. The expression of KAT8 of EV and *Kat8*^*OE*^ was analyzed by western blotting. **B**–**E**. SOX2 (red), PAX6 (red) and DAPI (blue) immunofluorescent staining of EV and *Kat8*^*OE*^ (**B**, **D**). Scale bar, 100 μm. The right panel shows the percentage of SOX2(**C**) and PAX6 (**E**) positive cells in two groups, n = 3. **F**–**I** TUJ1 (green), MAP2 (red) and DAPI (blue) immunofluorescent staining of EV and *Kat8*^*OE*^ (**F**, **G**). Scale bar. 100 μm for top panels; 50 μm for bottom panels. The bottom panel shows the percentage of MAP2 and TUJ1 positive cells (**H**, **I**) in two groups, n = 3. **J**. Model of the *Fto*/*Kat8* regulated axis and its influence over self-renewal and differentiation of CGNs during fetal cerebellar development. *, *P* < 0.05; **, *P* < 0.01. Student *t* test. All data are presented as the means ± SD. EV, empty vector. OE, overexpression. SD, standard deviation

To prove whether *Fto* loss affects *Kat8* and H4K16ac by regulating m^6^A modification, GNPs from exogenous mutations of the *Fto* functional domain were generated (*Fto*^*Mut*^), and the m^6^A levels were increased after *Fto*^*Mut*^ as shown by dot blot assay which was compared to *Fto* overexpression group (*Fto*^*OE*^) (Supplementary Fig. 8A). The IF results indicated that *Fto*^*Mut*^ restored the expression of the self-renewal genes *Sox2* and *Pax6* fully with regard to the *Fto*^*OE*^ group (Fig. 8A, B and Supplementary Fig. 8B, C). Following differentiation of GNPs into CGNs, *Fto*^*Mut*^ restored the expression of differentiation markers MAP2 and TUJ1 (Fig. 8C, D and Supplementary Fig. 8D, E). Furthermore, the m^6^A recognized protein IGF2BP3 knockdown (*Igf2bp3*^*KD*^)/*Fto*^*KD*^ GNPs were generated in the self-renewal and differentiation culture systems. The IF results indicated that *Igf2bp3*^*KD*^ restored the self-renewal genes *Sox2* and *Pax6* expression in the *Fto*^*KD*^ group (Fig. 8E, F and Supplementary Fig. 8F, G). In addition, when GNPs were differentiated into cerebellar CGNs, the IF results showed that Igf2bp3^KD^ fully restored the expression of the differentiation markers MAP2 and TUJ1 in the *Fto*^*KD*^ group (Fig. 8G, H and Supplementary Fig. 8H, I). To confirm whether a high level of *Kat8* influences cerebellar development, the *Kat8* overexpression GNPs were produced (*Kat8*^*OE*^) in Fig. 9A. The IF results indicated that *Kat8*^*OE*^ significantly inhibited the expression of self-renewal genes *Sox2* and *Pax6* (Fig. 9B–E). When differentiating into CGNs, *Kat8*^*OE*^ reduced the number of positive cells in differentiation markers MAP2 and TUJ1 observably (Fig. 9F-I).

## Discussion

Epitranscriptomic regulation, particularly m^6^A methylation, is becoming increasingly important in cerebellar development and function, according to new findings [11, 27]. It is unknown whether m^6^A demethylase FTO serves a specific function in the cerebellum. In this study, we demonstrate that *Fto*^*KO*^ mice exhibit cerebellar ataxia, including tremors and abnormal gait patterns. We show that knockout of *Fto* has a more severe impact on gene transcriptional and post-transcriptional regulation of cerebellum developmental mRNAs. Our results indicate crucial and distinct roles for *Fto* in cerebellar development.

The ability of FTO to eliminate m^6^A modifications from mRNA has an impact on the stability, translation, and splicing of transcripts, which play a critical role in brain development [28, 29]. Aberrant FTO activity has been linked to neurodevelopmental disorders, highlighting its importance in brain development and function [30, 31]. Moreover, the 6-methyladenine (6 mA) in genomic DNA mediated by *Fto* has also provided insights into development. Recent evidence reveals that FTO may influence 6 mA dynamics, thereby affecting developmental gene regulation [13, 32]. The interaction between 6 mA and m^6^A modifications mediated by FTO complicates our knowledge of gene regulation during ontogenesis. The study we conducted was the first to discover upregulation of *Kat8* caused by *Fto*^*KO*^ in an m^6^A-dependent manner (Fig. 3).

Recent study on the KAT8 complex, particularly its interaction with MSL (male-specific lethal) and NSL (non-specific lethal) complexes, has provided profound insights into its role in ontogeny through histone modification mechanisms [33]. KAT8, also known as MOF or MYST1, is a pivotal histone acetyltransferase that collaborates with the MSL and NSL complexes to regulate chromatin dynamics and gene expression during development [22]. The MSL complex, which includes KAT8, primarily mediates H4K16ac, a modification crucial for dosage compensation in Drosophila and chromatin regulation in other organisms [34]. This acetylation is essential for the maintenance of an open chromatin state, which facilitates the transcriptional activation of developmental genes. Another crucial participant, the NSL complex, interacts with KAT8 to establish and maintain active chromatin marks such as H4K16ac. The NSL complex’s role in histone modification and chromatin remodeling complements the function of the KAT8 complex by regulating gene expression at the level of enhancer-promoter interactions and maintaining global chromatin structure [35]. Additionally, the KAT8 complex’s association with other chromatin-modifying complexes, such as the SWI/SNF complex, has been linked to fine-tuning gene expression during ontogeny, highlighting the integrated nature of chromatin regulation [36]. In this study, we found for the first time that high H4K16ac modification levels promoted chromatin opening and activated cerebellar developmental gene expression in *Fto*^*KO*^ (Fig. 4 and 5). However, whether KAT8 regulates gene expression by forming MSL or NSL complexes still requires further verification. Furthermore, a separate study indicates H4K16ac activates the transcription of transposable elements and contributes to their cis-regulatory function [24]. Future studies should focus on whether H4K16ac regulates developmental processes in the cerebellum by affecting transposable elements.

Recent research has greatly advanced our understanding of the role of IGF2BPs in regulation of m^6^A methylation mechanisms during ontogenesis. It is noteworthy that our Co-IP experiments revealed that Kat8 binds to IGF2BP3 specifically, but not to IGF2BP1 or IGF2BP2. This may also explain why the knockdown of *Igf2bp3* completely restored related genes in the cerebellum (*Tuj1*, *Pax6*, *Sox2* and *Map2*). IGF2BP3, a member of the IGF2BP family (IGF2BP1-3) of RNA-binding proteins, functions as a critical reader of m^6^A modifications, regulating several aspects of RNA metabolism such as stability, localization, and translation [37]. Mutations in *Igf2bp3’s* KH or RRM domains have been shown to interfere with its ability to detect m^6^A [38]. IGF2BP3 proteins primarily recognize specific RNA sequences rich in GGAC motifs or similar sequences in the 3' untranslated regions (3'UTRs) or coding sequences (CDS) of mRNAs [39]. It stabilizes these mRNAs, enhancing their translation and contributing to cell differentiation and development [18, 40]. This role is particularly important during embryonic development, where precise regulation of gene expression is crucial for correct ontogenesis [41]. Instead, for YTHDF family proteins, the YTH domain is dedicated to identifying and binding to m^6^A-modified RNA [42]. Previous reports revealed that mutations in *Ythdf2* significantly affect its binding to the conserved G (m^6^A) C core motif, which is typically recognized by the YTH domains located at the C-terminus of YTHDF2 [43]. In recent years, studies have elucidated the impact of IGF2BP3 on stem cell biology and differentiation [44]. Furthermore, investigations have demonstrated that readers can activate gene expression by binding the histone modification enzyme [45, 46]. In our study, we found IGF2BP3 recognized the m^6^A modification on *Kat8* mRNA to increase its expression and further increased the level of histone H4K16ac modification by recruiting KAT8 (Fig. 9J). Previous study reported that *Atoh1* deletion disrupts granule neuron precursors proliferation and induces differentiation in the EGL region [47]. Meanwhile, *Atoh1* as a transcription factor can bind to a 10-nucleotide motif to directly regulate genes involved in migration, cell adhesion, metabolism during cerebellar development [48]. Besides, altered *Atoh1* levels in the hypoxic neonatal brain affected neuronal development and microtubule stability [49]. *Atoh1* is a master transcription factor critical for GNP proliferation and differentiation. Our results indicate that the deletion of *Fto* reduces the transcriptional level and protein expression level of the *Atoh1* gene in the cerebellar tissue. Changes in H4K16ac modification or alterations in chromatin accessibility were observed in the *Atoh1* promoter region in the CUT&Tag-seq or ATAC-seq datasets of the fetal cerebellum. Future studies should focus on the specific mechanisms of *Igf2bp3*-mediated regulation and its broader implications for cerebellar development and function. Understanding the specific molecular pathways and interactions influenced by *Igf2bp3* will help us understand how this protein contributes to the growth and maturation of cerebellar cells. Additionally, exploring the impact of *Igf2bp3* on overall cerebellar function could uncover its role in neurological health and disease, potentially leading to novel therapeutic approaches for cerebellar disorders.

## Conclusions

In summary, our findings first shed fresh light on the regulatory functions of *Fto* in the development of the prenatal mouse cerebellum, particularly its interactions with the histone-modifying enzyme KAT8. Upregulation of *Kat8* caused by *Fto*^*KO*^ in an m^6^A-dependent manner led to increased recruitment of KAT8 by IGF2BP3, which improved chromatin accessibility and H4K16ac modification, facilitating cerebellar developmental dysfunction progression. The discovery of the *Fto*-*Kat8*-*Igf2bp3*-H4K16ac axis sheds new insights on how RNA modifications influence chromatin states and gene expression during critical phases of cerebellar development.

## Supplementary Information

Supplementary file 1.

## Data Availability

All data generated or analyzed during this study are included in this manuscript and its supplementary information files. All reagents in this study are available. The key reagents are listed in Table S4.

## Abbreviations

3'UTRs: 3' Untranslated regions
6 mA: 6-Methyladenine
ATAC-seq: Assay for transposase-accessible chromatin with high throughput sequencing
CDS: Coding sequences
CGCs: Cerebellar granule cells
CGNs: Cerebellar granule neurons
Co-IP: Co-immunoprecipitation
CUT&Tag-seq: Cleavage under targets and tagmentation
DAPI: 4,6-Diamidino-2-phenylindole dihydrochloride
E13.5: Embryonic day 13.5
EGL: External granule cell layer
EV: Empty vector
GCPs: Granule cell progenitors
GCs: Granule cells
GNPs: Granule neuron precursors
GO: Gene ontology
GSEA: Gene set enrichment analysis
IF: Immunofluorescence
IGL: Internal granule cell layer
IGV: Integrative genomics viewer
KD: Knockdown
KEGG: Kyoto encyclopedia of genes and genomes
KO: Knockout
m^6^A: *N*^6^-Methyladenosine
m^6^A-RIP-seq: M^6^A RNA immunoprecipitation sequencing
MB: Methylene blue
MACS2: Model -based analysis for ChIP-Seq
ML: Molecular layer
MSL: Male-specific lethal
MUT: Mutant
NSCs: Neural stem cells
NSL: Non-specific lethal
OE: Overexpression
P3: Postnatal day 3
P7: Postnatal day 7
PCL: Purkinje cell layer
RL: Rhombic lip
RT: Room temperature
SD: Standard deviation
TEs: Transposable elements
TSS: Transcription start site
TST: Tail-suspension test
TUNEL: Terminal uridine deoxynucleotidyl transferase (dUTP) nick end labeling
VZ: Ventricular zone
WT: Wild type
