# Supplementary material for: Fto-mediated m6A modification is essential for cerebellar development through regulating epigenetic reprogramming
Source: J Biomed Sci. 2025 Aug 29;32:81. doi: 10.1186/s12929-025-01176-0 (PMC12398073; doi:10.1186/s12929-025-01176-0)
Supplement: Supplementary file 1 [file 12929_2025_1176_MOESM1_ESM.docx]

**Fto-mediated m6A modification is essential for cerebellar development through regulating epigenetic reprogramming**

**Supplementary figure legends**

**
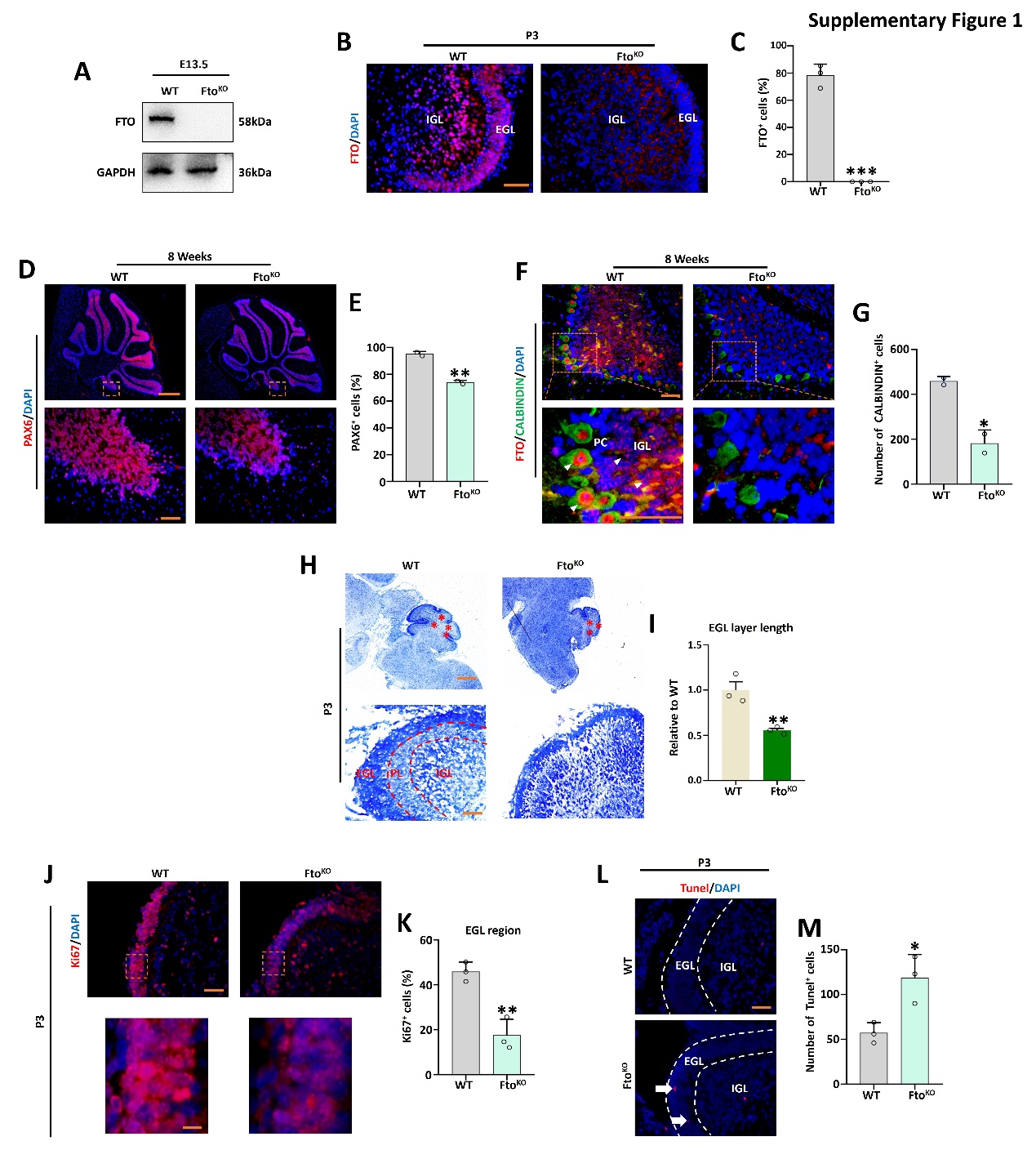
**

**Supplementary Fig. 1. Loss of *Fto* consistently affected cerebellar development and maturation.**

**A.** The expression of FTO in E13.5 cerebellar tissues of WT and *Fto^KO^* mice was analyzed by western blotting.

**B, C.** FTO (red) and DAPI (blue) immunofluorescent staining of WT and *Fto^KO^* mice cerebellum at P3 (B). Scale bar, 50 μm. The right panel shows the percentage of FTO positive cells (C), n = 3.

**D, E.** PAX6 (red) and DAPI (blue) immunofluorescent staining of WT and *Fto^KO^* mice cerebellum at 8 Weeks (D). Scale bar, 500 μm for top panels; 50 μm for bottom panels. The right panel shows the percentage of PAX6 positive cells (E), n = 2.

**F, G.** FTO (red), Calbindin (green) and DAPI (blue) immunofluorescent staining of WT and *Fto^KO^* mice cerebellum at 8 Weeks (F). Scale bar, 50 μm. The right panel shows the number of Calbindin positive cells (G), n = 2.

**H, I.** Nissl-stained sagittal cerebellar sections from WT and *Fto^KO^* mice cerebellum at P3 (H). Scale bar, 500 μm for top panels; 50 μm for bottom panels. The right panel shows the EGL thickness of the cerebellum at P3 (I), n = 3.

**J, K.** Immunofluorescence analysis of P3 cerebellar paraffin sections using a Ki67 antibody (red) and DAPI (blue). Scale bar, 50 μm. The right panels show the percentage of Ki67 positive cells in WT and *Fto^KO^* mice (K), n = 3.

**L, M.** TUNEL analysis of P3 cerebellar paraffin sections (L). Scale bar, 50 μm. The right panels show the number of TUNEL positive cells in WT and *Fto^KO^* mice (M), n = 3.

*, *P* < 0.05; **, **P** < 0.01; ***, *P* < 0.001. E13.5. embryonic day 13.5; P3. postnatal day 3; IGL. internal granule cell layer; EGL. external granule cell layer; PC. Purkinje cell layer; SD. standard deviation.


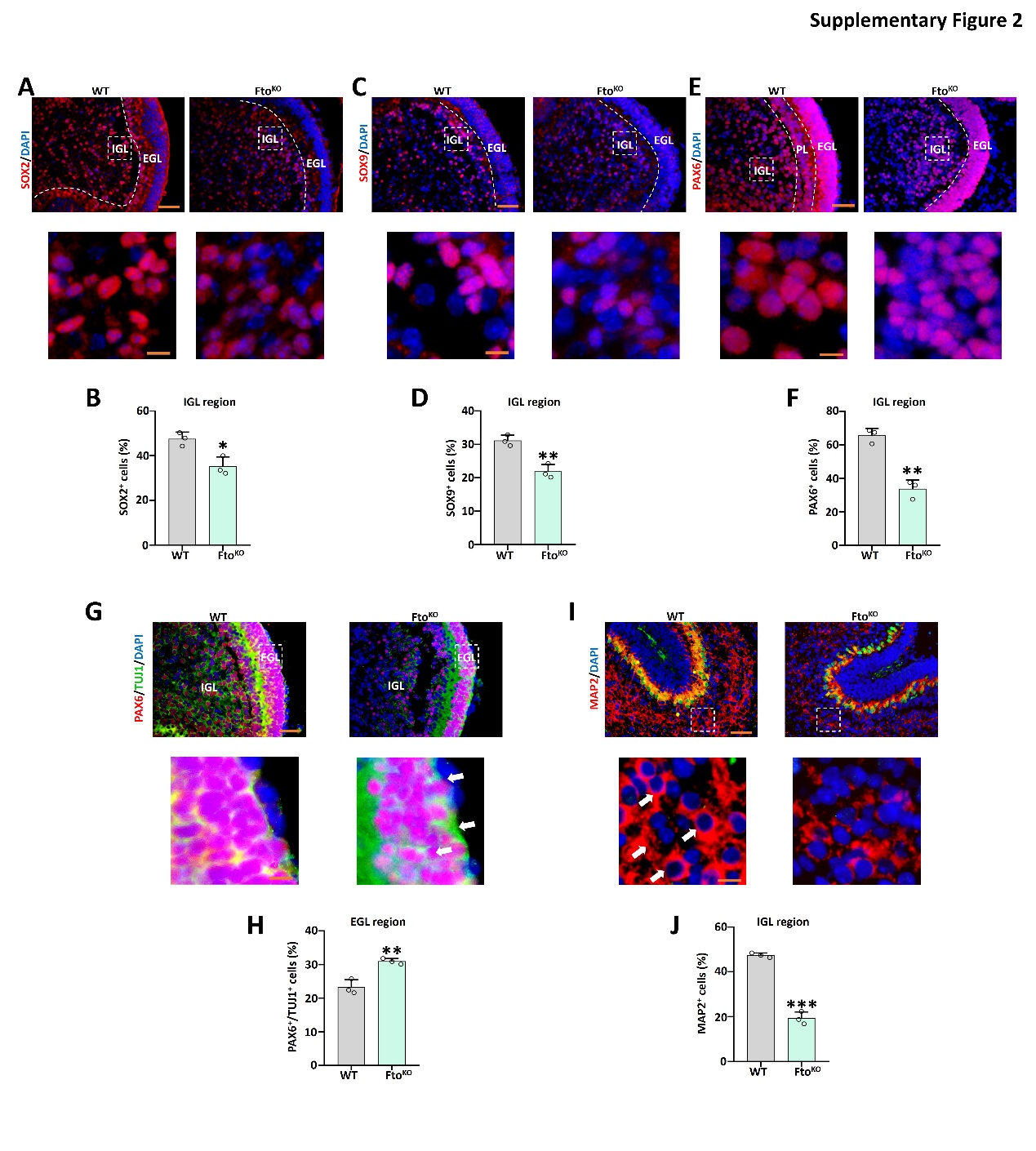


**Supplementary Fig. 2. Loss of the Fto gene in mice will trigger the cerebellar neurons dysplasia at P3.**

**A, B.** Immunofluorescence analysis of P3 cerebellar paraffin sections using a SOX2 antibody (red) and DAPI (blue). Scale bar, 50 μm. The bottom panels show the percentage of SOX2 positive cells in IGL region (B), n = 3.

**C, D.** Immunofluorescence analysis of P3 cerebellar paraffin sections using a SOX9 antibody (red) and DAPI (blue). Scale bar, 50 μm. The bottom panels show the percentage of SOX9 positive cells in IGL region (D), n = 3.

**E, F.** Immunofluorescence analysis of P3 cerebellar paraffin sections using a PAX6 antibody (red) and DAPI (blue). Scale bar, 50 μm. The bottom panels show the percentage of PAX6 positive cells in IGL region (F), n = 3.

**G, H.** PAX6 (red), TUJ1 (green) and DAPI (blue) immunofluorescent staining of WT and *Fto^KO^* mice cerebellum at P3 (G). Scale bar, 50 μm. The bottom panels show the percentage of double-positive PAX6 and TUJ1 cells in EGL region (H), n = 3.

**I, J.** Immunofluorescence analysis of P3 cerebellar paraffin sections using a MAP2 antibody (red) and DAPI (blue). The bottom panels show the percentage of MAP2 positive cells in IGL region (J), n = 3. Scale bar, 50 μm.

*, *P* < 0.05; **, *P* < 0.01; ***, *P* < 0.001. Student *t* test. All data are presented as the means ± SD. P3. postnatal day 3; IGL. internal granule cell layer; EGL. external granule cell layer. SD. standard deviation.


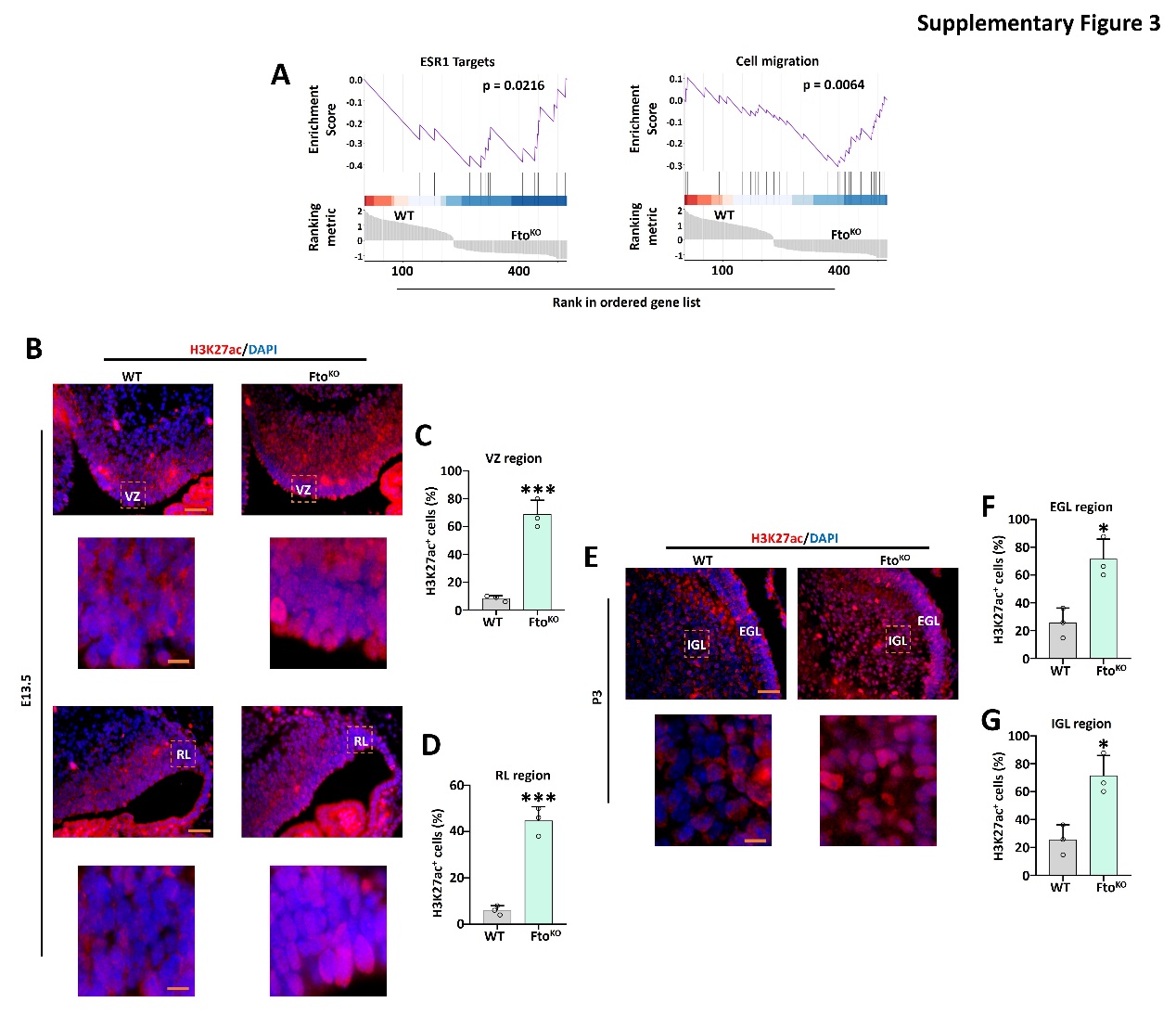


**Supplementary Fig. 3.** ***Fto*-mediated m^6^A modification influenced fetal cerebellar development through histone modification.**

**A.** The GSEA method showing that m^6^A differential genes were enrichment in the ESR1 Targets and cell migration.

**B-D.** H3K27ac (red) and DAPI (blue) immunofluorescent staining of WT and *Fto^KO^* mice cerebellum at E13.5 (B). Scale bar, 50 μm. The right panels show the percentage of H3K27ac positive cells in VZ and RL region (C, D) of two groups, n = 3.

**E-G.** H3K27ac (red) and DAPI (blue) immunofluorescent staining of WT and *Fto^KO^* mice cerebellum at P3 (E). Scale bar, 50 μm. The right panels show the percentage of H3K27ac positive cells in EGL and IGL region (F, G) of two groups, n = 3.

*, *P* < 0.05; **, *P* < 0.01; ***, *P* < 0.001. Student *t* test. All data are presented as the means ± SD. E13.5. embryonic day 13.5; P3. postnatal day 3; VZ. ventricular zone; RL. rhombic lip; IGL. internal granule cell layer; EGL. external granule cell layer. SD. standard deviation.


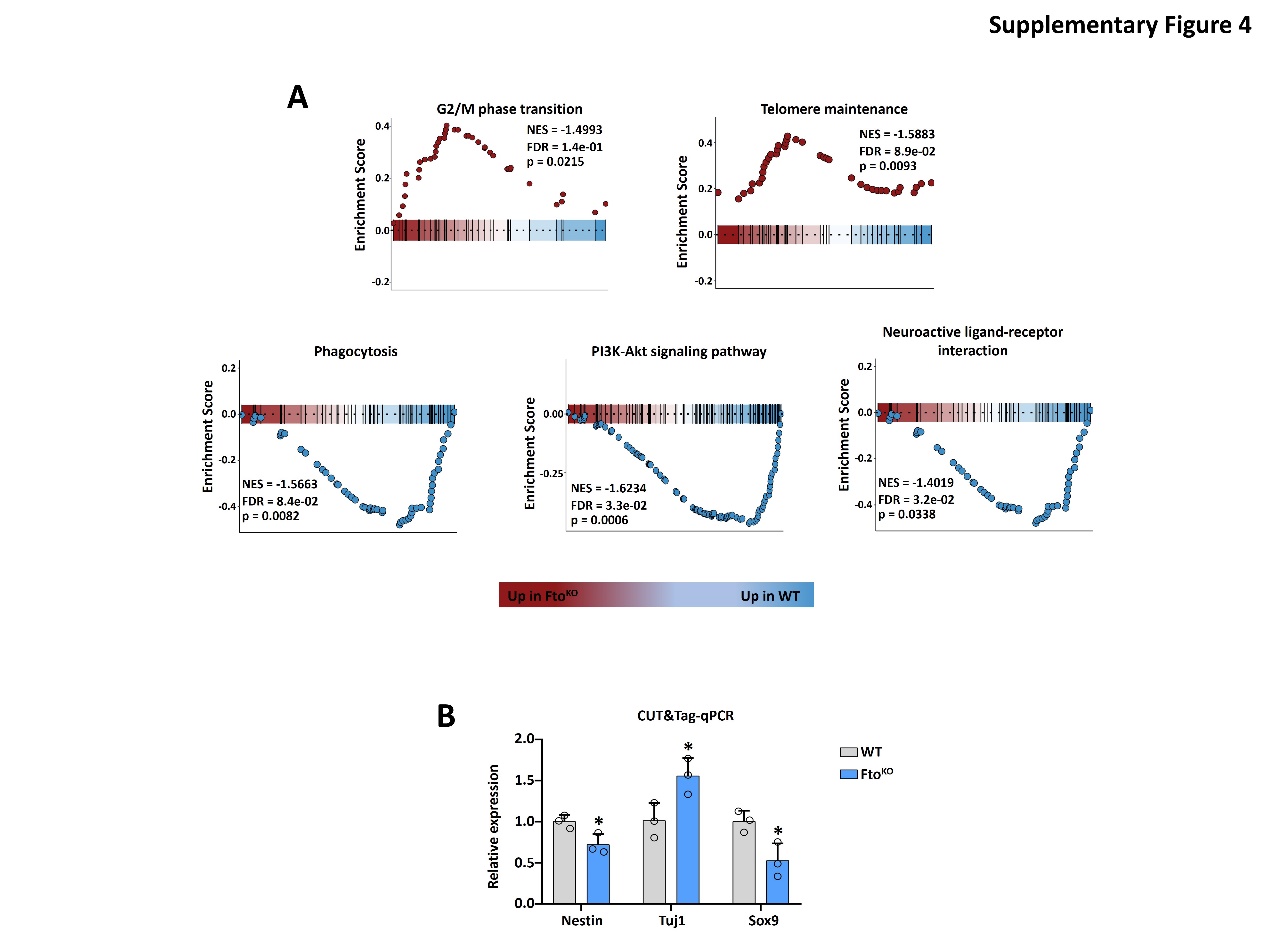


**Supplementary Fig. 4. GSEA and CUT&Tag-qPCR method accessed the H4K16ac enrichment pathway and genes after *Fto^KO^* in cerebellum.**

**A.** GSEA assay showed that *Fto* deletion affecting H4K16ac enriched genes were related to G2/M phase transition, telomere maintenance, phagocytosis, PI3K-Akt signaling pathway and neuroactive ligand-receptor interaction.

**B.** CUT&Tag-qPCR showing the level of H4K16ac modification on the neural differentiation and self-renew genes in WT and *Fto^KO^* mice at E13.5 (*Nestin*, *Tuj1*, *Sox9*).


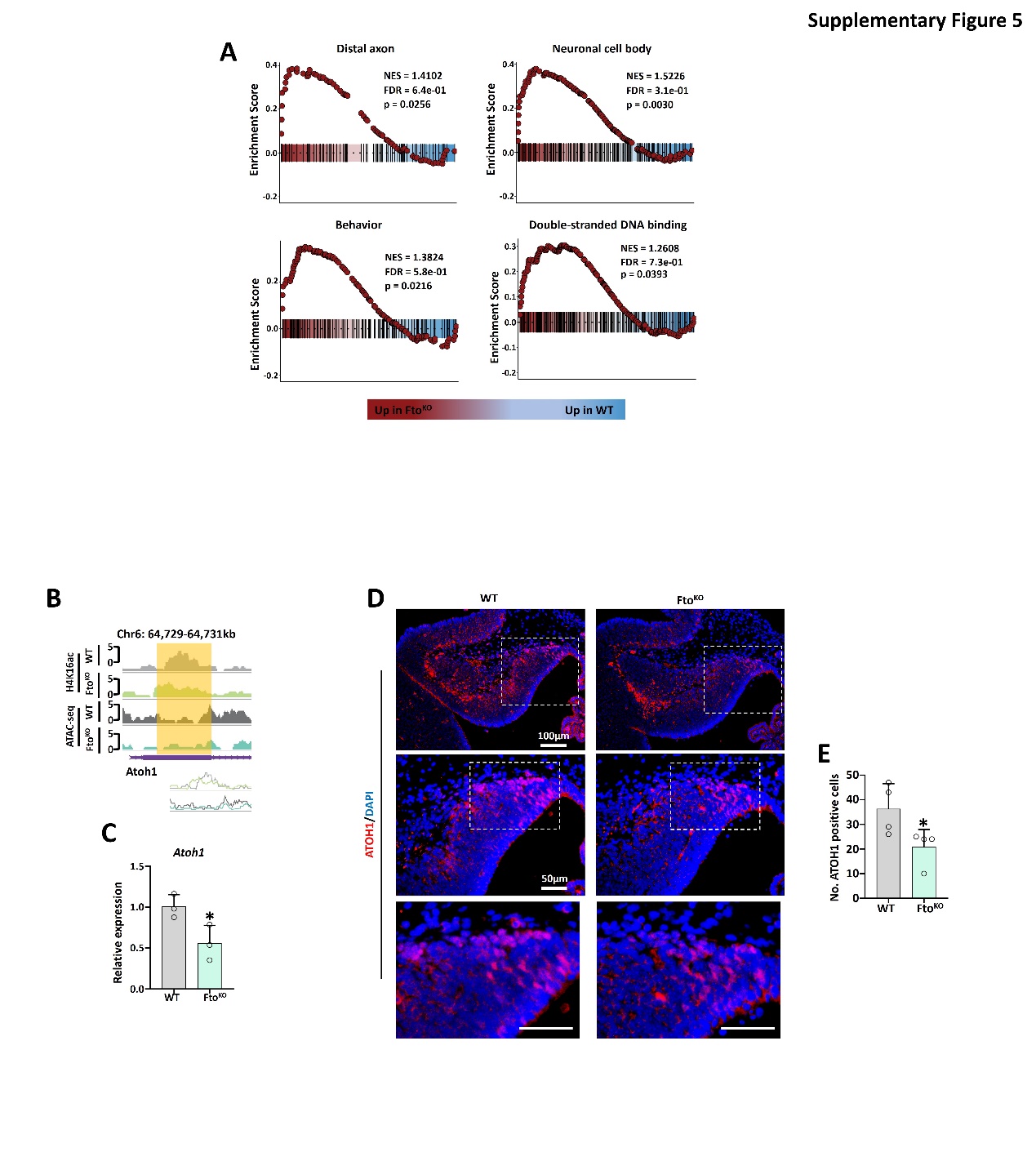


**Supplementary Fig. 5.** ***Fto^KO^* regulated neurological function through affecting chromatin accessibility.**

**A.** GSEA assay showed that *Fto* deletion affecting chromatin enriched genes were related to double-stranded DNA binding, neuronal cell body, behavior, distal axon.

**B.** IGV demonstrated distinct enrichment in H4K16ac and chromatin in the fetal cerebellar function of regulatory genes *Atoh1* promoter regions. n=2.

**C.** mRNA level of *Atoh1* was reduced after *Fto* deletion in the cerebellar tissues at E13.5. n=3.

**D, E.** ATOH1 (red) and DAPI (blue) immunofluorescent staining of WT and *Fto^KO^* mice cerebellum at E13.5 (D). Scale bar, 100 μm for top panels; 50 μm for middle and bottom panels. The right panel shows the number of ATOH1 positive cells (E), n = 4.**
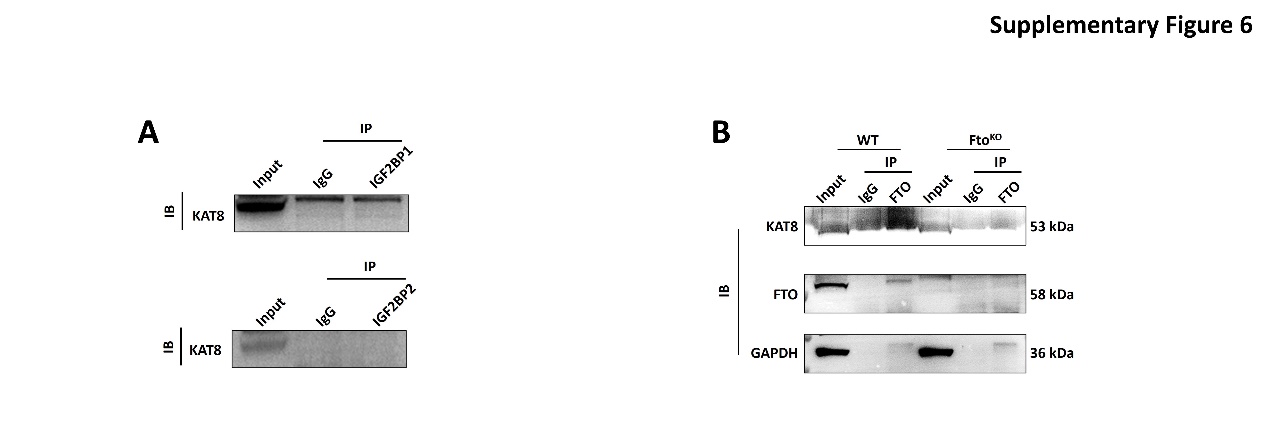
**

**Supplementary Fig. 6. The protein interaction between KAT8 and IGF2BP1/2**

**A.** Co-immunoprecipitation of IGF2BP1 or IGF2BP2 from mouse cerebellums lysates, followed by immunoblot of KAT8. IgG was used as a negative control.

**B.** Co-immunoprecipitation of FTO in *Fto^KO^* and WT group, followed by immunoblot of KAT8 and FTO. IgG was used as a negative control.

**Supplementary Figure 7**


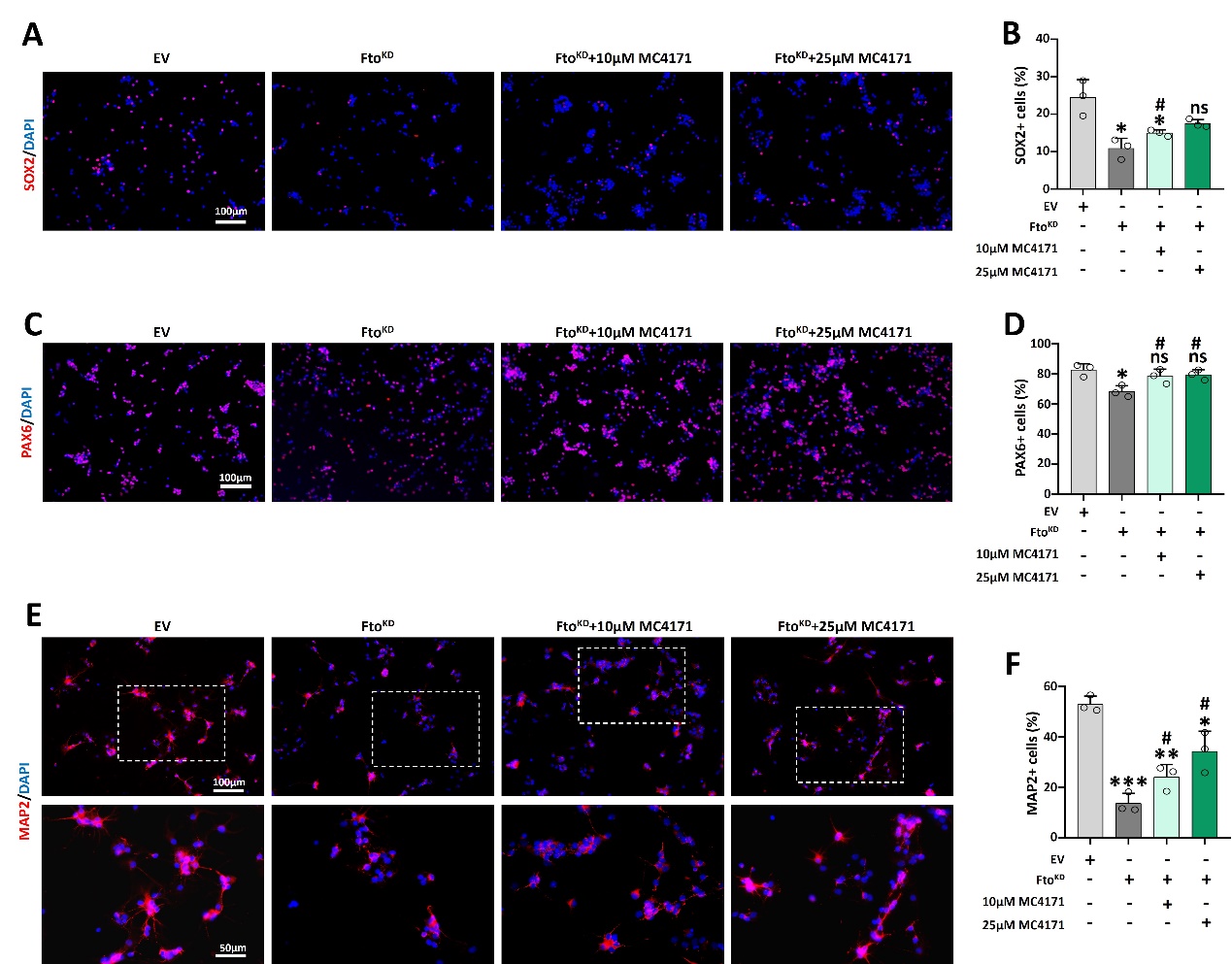


**Supplementary Fig. 7. H4K16AC modification affected neural self-renewal and differentiation.**

**A, B.** SOX2 (red) and DAPI (blue) immunofluorescent staining of EV, *Fto^KD^*, *Fto^KD^*+10μM MC4171 and *Fto^KD^*+25μM MC4171 groups(A). Scale bar, 100 μm. The right panel shows the percentage of SOX2 positive cells (B) in three groups, three biological replicates.

**C, D.** PAX6 (red) and DAPI (blue) immunofluorescent staining of EV, *Fto^KD^*, *Fto^KD^*+10μM MC4171 and *Fto^KD^*+25μM MC4171 groups(C). Scale bar, 100 μm. The right panel shows the percentage of PAX6 positive cells (D) in three groups, three biological replicates.

**E, F.** MAP2 (red) and DAPI (blue) immunofluorescent staining of EV, *Fto^KD^*, *Fto^KD^*+10μM MC4171 and *Fto^KD^*+25μM MC4171 groups(E). Scale bar, 100 μm for top panels; 50 μm for bottom panels. The right panel shows the percentage of MAP2 positive cells (F) in three groups, three biological replicates.

Student *t* test. *, P < 0.05; **, P < 0.01; ***, P < 0.001, ns = no significant, compared with EV group. #, P < 0.05; compared with *Fto^KD^* group. All data are presented as the means ± SD. SD. standard deviation.


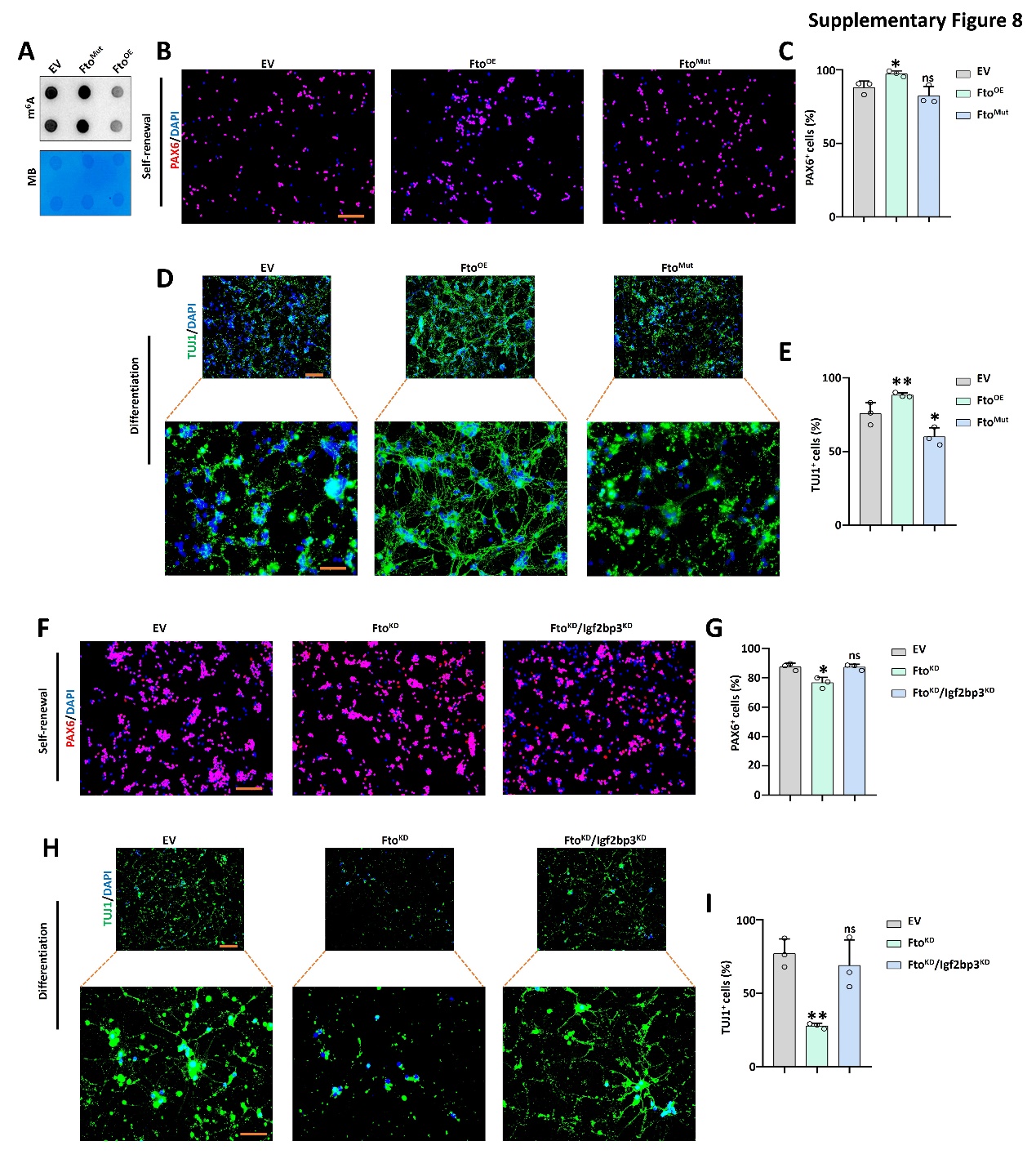


**Supplementary Fig. 8. *Fto*-mediated m^6^A modification affected neural self-renewal and differentiation.**

**A.** Dot Blot detected the m^6^A level in the EV, *Fto^OE^* and *Fto^Mut^* groups. RNA spots stained with methylene blue (MB) were used as a reference.

**B, C.** PAX6 (red) and DAPI (blue) immunofluorescent staining of EV, Fto^OE^ and Fto^Mut^ groups(B). Scale bar, 100 μm. The right panel shows the percentage of PAX6 positive cells (C) in three groups, three biological replicates.

**D, E.** TUJ1 (green) and DAPI (blue) immunofluorescent staining of EV, *Fto^OE^* and *Fto^Mut^* (D). Scale bar, 100 μm for top panels; 50 μm for bottom panels. The right panel shows the percentage of TUJ1 positive cells (E) in three groups, three biological replicates.

**F, G.** PAX6 (red) and DAPI (blue) immunofluorescent staining of EV, *Fto^KD^* and *Fto^KD^*/*Igf2bp3^KD^* groups(F). Scale bar, 100 μm. The right panel shows the percentage of PAX6 positive cells (G) in three groups, three biological replicates.

**H, I.** TUJ1 (green) and DAPI (blue) immunofluorescent staining of EV, *Fto^KD^* and *Fto^KD^*/*Igf2bp3^KD^* (H). Scale bar, 100 μm for top panels; 50 μm for bottom panels. The right panel shows the percentage of TUJ1 positive cells (I) in three groups, three biological replicates.

*, *P* < 0.05; **, *P* < 0.01. Student *t* test. All P-values are compared with the EV group. All data are presented as the means ± SD. CGCs. cerebellar granule cells; SD. standard deviation.

**Materials**

**Table S1.** **The primers used for genotyping were studied**

| **Gene** | **Forward sequence(5′-3′)** | **Reverse sequence(5′-3′)** |
| --- | --- | --- |
| **Fto** | **ccagtgtctcgcatcctcatc** | **TTACTCATCCTCAGAGCCTCAGA** |

**Table S2. Designations, sequences of shRNA and plasmid amplicons**

| **Name** | **Sequence from 5'-3'** |
| --- | --- |
| **shNC** | **GGTTCTCCGAACGTGTCACGT** |
| **shFto (M)** | **GATGATGAAGTGGACCTTAAG** |
| **Kat8^KD^(M)** | **CTCATGTGCAGAAGACCTATG** |
| **Igf2bp3^KD^(M)** | **GCAGTTGTAAATGTAACCTAT** |
| **Empty vector(M)** | **pcDNA3.1-3xFlag** |
| **Fto^OE^(M)** | **GenBank Accession No. NP_036066.2** |
| **Fto^Mut^-F(M)** | **CTGGGCTCACGCTGAGAACCTGGTGGACAGGTCA** |
| **Fto^Mut^-R(M)** | **TTCTCAGCGTGAGCCCAGCTCACCGCCATCTTC** |
| **Kat8^OE^(M)** | **GenBank Accession No. NP_001347628.1** |

**Table S3 Key antibodies resources**

| **REAGENT or RESOURCE** | **SOURCE** | **IDENTIFIER** | **Antibodies used** |
| --- | --- | --- | --- |
| **Rabbit anti-m6A** | **Cell Signaling Technology** | **Cat# 56593S;**  **RRID:AB_2799515** | **Dot blot:1:3000**  **RIP 0.5μg** |
| **Rabbit anti-FTO** | **ProteinTech** | **Cat# 27226-1-AP;**  **RRID:** **AB_2880809** | **WB 1:1000,**  **IF 1:500,IP:4μg** |
| **Rabbit anti-IGF2BP3** | **ProteinTech** | **Cat# 14642-1-AP;**  **RRID:AB_2122782** | **WB 1:2000,**  **IP:4μg** |
| **Rabbit anti- Ki67** | **Abcam** | **Cat# AB15580;**  **RRID:AB_443209** | **IF 1:1000** |
| **Rabbit anti-SOX2** | **ProteinTech** | **Cat# 11064-1-AP;**  **RRID:** **AB_2195801** | **IF 1:500** |
| **Rabbit anti-SOX9** | **Millipore** | **Cat# AB5535;**  **RRID: AB_2239761** | **IF 1:500** |
| **Rabbit anti-PAX6** | **ProteinTech** | **Cat# 12323-1-AP;**  **RRID: AB_2159695** | **IF 1:500** |
| **Mouse anti-Nestin** | **Abcam** | **Cat# ab6142;**  **RRID: AB_305313** | **IF 1:500** |
| **Rabbit anti-Nestin** | **ProteinTech** | **Cat# 19483-1-AP;**  **RRID: AB_10644440** | **IF 1:500** |
| **Mouse anti-TUBB3** | **ProteinTech** | **Cat# 66375-1-Ig;**  **RRID: AB_2814998** | **IF 1:500** |
| **Mouse anti-MAP2** | **Abcam** | **Cat# ab183830；RRID:AB_2895301** | **IF 1:500** |
| **Mouse anti-Calbindin D28K** | **SANTA CRUZ** | **Cat# 26497-1-AP;**  **RRID: AB_2880533** | **IF 1:500** |
| **Rabbit anti-H4K16ac** | **Abcam** | **Cat# ab109463;**  **RRID: AB_10858987** | **IF 1:2000，5ug per CUT&Tag** |
| **Rabbit anti-KAT8** | **ProteinTech** | **Cat# 13842-1-AP;**  **RRID:** **AB_2146894** | **IB 1:1000**  **IP:4μg** |
| **Rabbit anti-KAT8** | **ABclonal** | **Cat# A2208;**  **RRID:** **AB_2764225** | **IF 1:500** |
| **Rabbit anti-H3K27ac** | **Abcam** | **Cat# ab177178;**  **RRID: AB_2828007** | **IF 1:2000,** |
| **Mouse anti-GAPDH** | **ProteinTech** | **Cat# 60004-1-Ig;**  **RRID: AB_2107436** | **WB 1:10000** |
| **Alexa Fluor 488 anti-Mouse** | **ThermoFisher** | **Cat#** **A21202;**  **RRID: AB_141607** | **IF 1:500** |
| **Alexa Fluor 555 anti-Rabbit** | **ThermoFisher** | **Cat#** **A31572;**  **RRID: AB_162543** | **IF 1:500** |
| **HRP*Goat Anti Rabbit IgG(H+L)** | **ProteinTech** | **Cat# RGAR001;**  **RRID:** **AB_3068333** | **WB 1:10000** |
| **HRP* Goat Anti Mouse IgG(H+L)** | **ProteinTech** | **Cat# RGAM001;**  **RRID:** **AB_3068333** | **WB 1:10000** |
| **IPKine™ HRP, Mouse Anti-Rabbit IgG LCS** | **Abbkine** | **Cat# A25022;**  **RRID:** **AB_2893334** | **WB 1:10000** |
| **Normal Rabbit IgG Control** | **R&D Systems** | **Cat# AB-105-C;**  **RRID:AB_354266** | **IP:4μg** |

**Table S4 Key reagent**

| **REAGENT or RESOURCE** | **SOURCE** | **IDENTIFIER** |
| --- | --- | --- |
| **0.25% Trypsin-EDTA** | **Gibco** | **Cat#25200072** |
| **Mouse cerebellar granule cell complete culture-medium** | **ZQXZBIO** | **Cat# PCM-M-83** |
| **DMEM Media** | **Gibco** | **Cat#C11995500CP** |
| **Fetal bovine serum** | **Gibco** | **Cat# 10099141C** |
| **Penicillin streptomycin -** | **Gibco** | **Cat# 15140122** |
| **Nissl Staining** **Solution** | **Servicebio** | **Cat#G1036** |
| **100μm Cell Strainer** | **Falcon®** | **Cat#352360** |
| **Poly-L-lysine Solution** | **Sangon Biotech** | **Cat# E607015** |
| **Triton X-100** | **Sigma** | **Cat# 93443; CAS# 9036-19-5** |
| **DAPI** | **Beyotime** | **Cat# C1005** |
| **Lipofectamin 2000** | **Invitrogen** | **Cat#** **11668019** |
| **Skim milk** | **BD** | **Cat# 232100** |
| **DTT** | **Invitrogen** | **Cat#** **772590** |
| **RIP****A buffer** | **Beyotime** | **Cat# P0013B** |
| **Phosphatase and Protease Inhibitor Cocktails** | **Roche** | **Cat#11873580001** |
| **BCA Protein Assay Kit** | **Beyotime** | **Cat#p0012** |
| **RNase inhibitor** | **TaKaRa** | **Cat#** **2313B** |
| **Maxima H Minus** | **Thermo Scientific** | **Cat#** **EP0752** |
| **Hybond N+ membranes** | **GE Healthcare** | **Cat#** **RPN303C** |
| **PVDF membrane** | **Millipore** | **Cat#** **IPVH00010** |
| **Protease inhibitor** | **Roche** | **Cat#****11873580001** |
| **In Situ Cell Death Detection Kit TMR red** | **Roche** | **Cat#12156792910** |
| **RNeasy MinElute Cleanup Kit** | **QIAGEN** | **Cat#74204** |
| **SMARTer Stranded Total RNA-Seq Kit v3 Pico Input Mammalian** | **TaKaRa** | **Cat#** **634488** |
| **Protein A Dynabeads** | **Thermo Scientific** | **Cat# 10002D** |
| **Protein G Dynabeads** | **Thermo Scientific** | **Cat# 10004D** |
| **Protein G-Agarose** | **Roche** | **Cat#11243233001** |
| **Qubit RNA HS Assay** | **Thermo Scientific** | **Cat# Q32852** |
| **Equalbit 1x dsDNA HS Assay Kit EQ121** | **Vazyme** | **Cat#** **EQ121-02** |
| **NP-40 Surfact-Amps** | **Thermo Scientific** | **Cat#** **28324** |
| **Na-deoxycholate** | **Sigma** | **Cat#** **D6750** |
| **CUT&Tag Kit** | **Vazyme** | **Cat# TD903-01** |
| **ATAC-Seq Library Prep Kit** | **Vazyme** | **Cat# TD711-02** |
| **β-mercaptoethanol** | **Sigma** | **Cat# 63689** |
| **RNAiso Plus** | **Takara** | **Cat# 9109** |
| **Turbo DNAase** | **Invitrogen** | **Cat# BL698A** |

**Software and algorithms**

| **Image J** | **NIH** | **https://imagej.nih.gov/ij/** |
| --- | --- | --- |
| **GraphPad Prism** | **GraphPad Prism9 Software, La Jolla California USA** | **https://www.graphpad.com** |
